# The search for, and chemistry and mechanism of, neurotrophic natural products

**DOI:** 10.1007/s11418-020-01431-8

**Published:** 2020-07-08

**Authors:** Yoshiyasu Fukuyama, Miwa Kubo, Kenichi Harada

**Affiliations:** Faculty of Pharmaceutical Sciences, Tokushima Bunri University, Tokushima, 770-8514 Japan

**Keywords:** Neurotrophin-mimic natural compound, Neurogenesis, Neurite outgrowth, *Magnolia ovobata*, *Illicium* species, *Viburnum* species, *Garcinia subelliptica*, *Phytolacca americana*

## Abstract

**Abstract:**

Neurotrophic factors, now termed neurotrophins, which belong to a class of polypeptidyl agents, have been shown to potentially be beneficial for the treatment of neurodegenerative diseases such as Alzheimer’s disease, because endogenous neurotrophic factors (NGF, BDNF, NT3, NT4) have been recognized to play critical roles in the promotion of neurogenesis, differentiation, and neuroprotection throughout the development of the central nervous system. However, high-molecular weight proteins are unable to cross the blood–brain barrier and are easily decomposed by peptidase under physiological conditions. To address this issue, small molecules that can mimic the functions of neurotrophic factors would be promising alternatives for the treatment of neurodegenerative disease. We have continued to search for natural products having typical neurotrophic properties, which can cause neurogenesis, enhance neurite outgrowth, and protect neuronal death using three cellular systems (PC12, rat cortical neurons, and MEB5 cells). In this review, we summarize the neurotrophic activities and synthesis of dimeric isocuparane-type sesquiterpenes from the liverwort, *Mastigophora diclados*, the mechanism of neurotrophic neolignans, magnolol, honokiol and their sesquiterpene derivatives, and introduce unique neurotrophin-mimic natural products, including *seco*-prezizaane-type sesquiterpenes from the *Illicium* species, vibsane-type diterpenes from *Viburnum awabuki*, and miscellaneous natural products with neurotrophic effects discovered by us.

**Graphic abstract:**

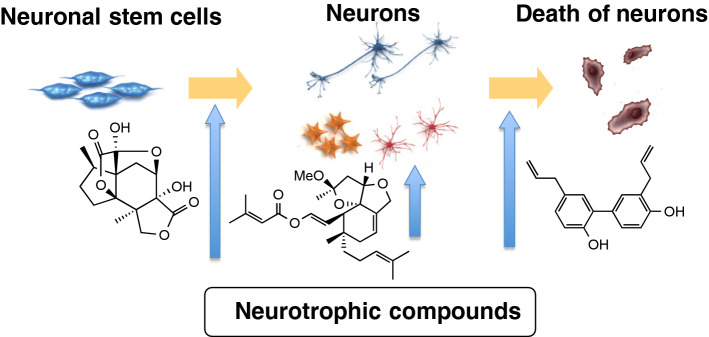

## Introduction

In recent years, the percentage of elderly people has increased. In Japan, the population ratio of people aged more than 65 years is estimated to reach 29.1% by 2020 and further increase to 38.5% by 2050 [1]. In a superaged society, people wish for healthy longevity and are eager for a fulfilling welfare society. On the other hand, with age, we suffer from various diseases, such as cardiovascular diseases, cancers, and dementia, and thus, it is essential to not only explore the etiology of these diseases but also develop therapeutic drugs and preventive methods.

In particular, the number of elderly individuals who suffer from senile dementia has increased through this superaged society. Senile dementias are regarded as neurodegenerative diseases, which are categorized as Alzheimer’s disease (AD), Parkinson’s disease (PD), Huntington’s disease (HD), and amyotrophic sclerosis, and are characterized by nervous system dysfunction resulting from progressive neuronal degeneration [2]. In Japan, the elderly population with neurodegenerative diseases will increase to 8,300,000 by 2030 unless suitable medical treatments are not realized [3]. AD is the most prevalent form of dementia, accounting for 50–56% of cases at autopsy and in clinical settings, and AD combined with intracerebral vascular diseases accounts for another 13–17% of cases.

The principle risk for AD is age. The incidence of AD doubles for every 5 years of age, but AD is not necessarily the outcome of aging [4]. The brain regions involved in learning and memory processes are reduced in size in AD patients as a result of degeneration of synapses and death of neurons [5]. It has been more than 15 years since it was first proposed that AD might be caused by deposition of amyloid β-peptide (Aβ) in plaques in the brain [6]. Accumulation of Aβ in the brain triggers the remaining AD pathogenesis, including the formation of neurofibrillary tangles containing tau protein, causing the degeneration of neurons and resulting in AD. Although tremendous efforts have been made according to the amyloid hypothesis, new drugs for the treatment of AD have not been successfully developed [7, 8]. This is presumably because the underlying pathogenesis of AD still remains to be explored [9].

It is well known that following neuronal injury, adult neurons have an intrinsic ability and dynamic repair mechanism within the central nervous system to regenerate and produce neuronal cells and restore neuronal networks, although this capacity is limited and the regions that are able to regenerate neurons are restricted [10]. From this perspective, we initiated our research project to discover small molecule natural products that have the potential to act as neurotrophins to enhance neurogenesis, promote neurite outgrowth, and protect the death of neurons. In this review, we will introduce our own research program on the basis of neurotrophic properties and then highlight neurotrophic natural products, in particular, focusing on the chemistry and biological profiles of our discovered active compounds.

## Neurotrophins and the screening system to search for neurotrophin mimetics

Neurotrophins (neurotrophic factors) have been shown to be potentially beneficial in the treatment of neurodegenerative diseases such as AD, Parkinson’s disease (PD) and Huntington’s disease (HD) because endogenous neurotrophic factors have been recognized to play critical roles in the promotion of neurogenesis, differentiation, and neuroprotection throughout the development of the central nervous system [10, 11]. In mammals, the known neurotrophins are nerve growth factor (NGF), brain-derived neurotrophic factor (BDNF), neurotrophin 3 (NT3), and neurotrophin 4 (NT4) [11]. These neurotrophins bind selectively to their tyrosine kinase receptors TrkA, TrkB and TrkC, and all of them bind non-selectively to the neurotrophin receptor p75, resulting in activation of neuronal signal transduction related to the broad spectrum of biological activities exerted by neurotrophins [12, 13]. Therapeutic uses of NFs by intracranial injections, transplantation of cells secreting NFs, or gene therapy have shown promising results in animal models of neuronal degeneration as well as in clinical trials [14–16]. However, as NFs are high-molecular weight proteins, they have been unable to cross the blood barrier and are easily decomposed by peptidase under physiological conditions. To address this issue, small molecules that can mimic the functions of neurotrophic factors would be promising alternatives for the treatment of neurodegenerative disease [17, 18].

**Fig. 1:**
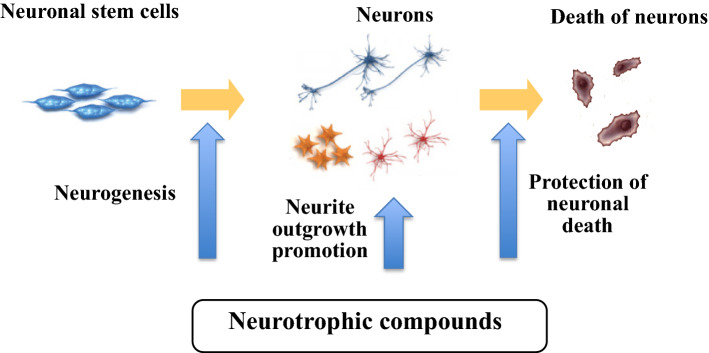
Protocol of searching for neurotrophic compounds by the assay system using three cells: PC12, primary cultured rat cortical neurons, and MEB5

Our protocol of searching for small molecules with neurotrophic properties is how to discovery NT mimicking compounds as well as to implicate active compounds in the key physiological functions of NTs: differentiation (neurogenesis), development (neurite outgrowth promotion) and survival (protection of neuronal death) of neurons [19] (Fig. 1). We applied three cells to the assay system; rat pheochromocytoma PC12 cells [20], primary cultured rat cortical neurons [21] and mouse multipotent neural precursor cells (MEB5) [22]. Both PC12 cells and NGF-mediated PC12 cells are used as the primary screening to identify active candidates. PC12 cells generate and extend neurites in response to NGF though the direct activation of the TrkA receptor or enhancing the intercellular NT signal pathway to induce neuritogenesis; whereas, NGF-mediated PC12 cells can extend the length of neurites to show neurite outgrowth through various mechanisms. Withdrawal of NGF from the culture medium causes the death of PC12 cells. This method is used to screen for protection against neuronal death. Primary cultured cortical neurons are used for the second screening to confirm neurite outgrowth promotion or protection of neuronal death under different culture conditions. Finally, MEB5 cell lines have been used to ascertain whether active compounds have the potential to induce the differentiation of stem cells into neurons. In this review, our chemical and biological studies on natural products with neurotrophic activity are compiled [23–25].

## Mastigophorenes: isocuparane-type sesquiterpene dimers from the liverwort *Mastigophora diclados*

**Fig. 2:**
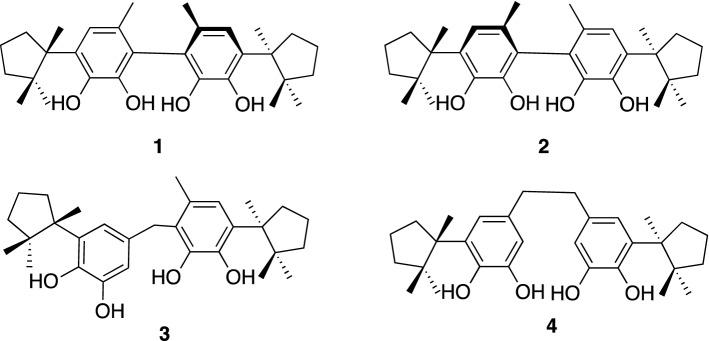
Mastigophorenes A (**1**), B (**2**), C (**3**) and D (**4**) from *Mastigophora diclados*

The liverworts elaborate a wide variety of terpenoids and lipophilic aromatic substances, which have been very often found to show different types of biological activity [26]. *M. diclados* (Brid.) Nees is a rather primitive liverwort and is commonly found in tropical Asiatic areas. Our independent study [27, 28] on the ether extract of *M. diclados* collected in Boruneo resulted in the isolation of four unique dimeric isocuparanes, mastigophorenes A (**1**), B (**2**), C (**3**), and D (**4**), together with their monomer unit, herbertenediol (**5**) [29] (Fig. 2). Mastigophorenes A (**1**), B (**2**), and D (**4**) were found to exhibit interesting neurotrophic properties at concentrations ranging from 0.1 to 10 μM, which could enhance neurite-sprouting and network formation in primary cell cultures derived from embryonic rat cerebral hemispheres [30]. On the other hand, mastigophorene C (**3**) and the monomeric compound, herbertenediol (**5**), suppressed the neurite outgrowth under the same conditions.

**Scheme 1:**
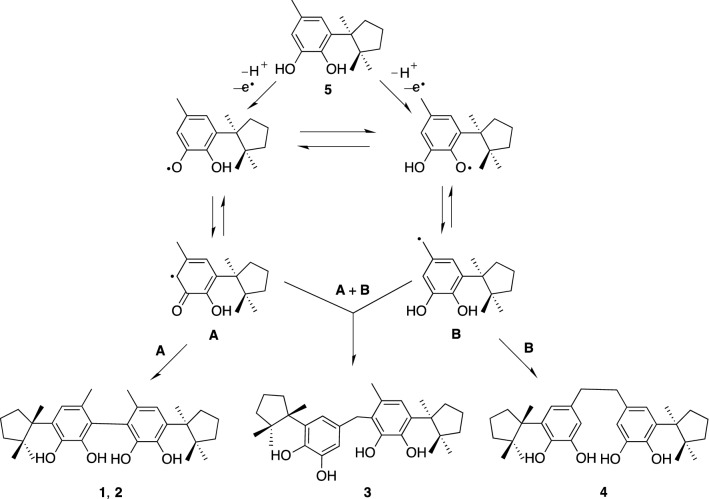
Biosynthetic route to dimeric isocuparenes **1**, **2**,** 3** and **4** based on one-electron oxidative coupling from (-)-herbertenediol (**5**)

**Fig. 3:**
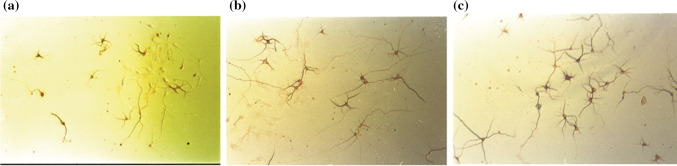
Enhancement of neurite outgrowth by mastigophorenes A (**1**) and B (**2**) in primary cultured rat cortical neurons in serum-free DMEM medium supplemented with B-27. **a** 0.5% EtOH, **b** 1μM mastigophorene A, **c** 1μM mastigophorene B

**Fig. 4:**
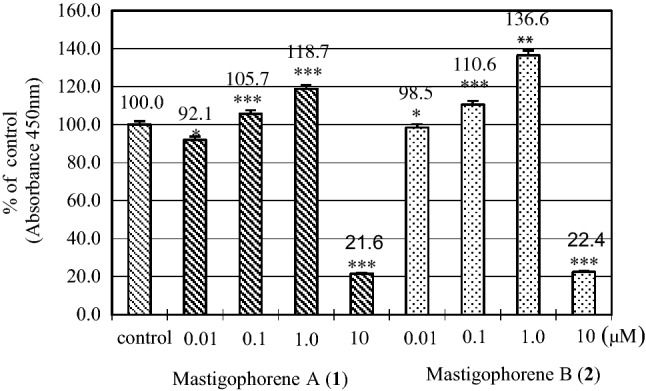
Neuroprotective activity of mastigophorenes A (**1**) and B (**2**) in primary cultured rat cortical neurons in serum-free DMEM medium supplemented with N-2. After the neuronal cells (2 × 10^5^ cells cm^−2^) were cultured for 3 days in the presence of 0.5% EtOH (control) and compounds **1** and **2**, neuronal viability was assessed by the WST-8 reduction assay. The data are expressed as means SE (*n* = 4); *p < 0.05, **p < 0.015, ***p < 0.005 versus control.

The dimeric compounds **1**–**4** could be derived from herbertenediol (**5**), a cometabolite in the plant, by phenolic oxidation. Compounds **1**–**4** are presumably biosynthesized via the phenoxy radical products formed from the one-electron oxidation of **5,** and then the formed radicals are subsequently converted into radical **A** or benzyl radical **B** which would give rise to a quinone methide via one more oxidation along with the loss of a proton radical. Homocoupling between two radicals **A** would lead to mastigophorenes A (**1**) and B (**2**) followed by aromatization; whereas, mastigophorene D (**4**) could be produced from the direct coupling between two benzyl radicals **B**. An alternative heterocoupling between radicals **A** and **B** would give rise to mastigophorene C (**3**) (Scheme 1) [28]. In fact, biomimetic oxidative coupling of 2-*O*-methylherbertenediol using (*tert-*BuO)_2_ in chlorobenzene under refluxing gave, after *O*-methyl deprotection with BBr_3_, two mastigophorenes A (**1**) and B (**2**) as an atrop-diastereomeric mixture (40:60) in 28% yield [31]. However, the direct oxidation of herbertenediol (**5**) with (*tert-*BuO)_2_ failed to yield dimers **1** and **2**, resulting in a complex mixture, and thus protecting the 1-hydroxy group of **5** was essential for successful oxidative coupling. We applied horseradish peroxidase (HRP)-catalyzed oxidative phenolic coupling to **5**, which resulted in the direct formation of **1** (10%) and **2** (18%) with recovery of **5** (72%) [32, 33]. With a large amount of synthesized **1** and **2** in hand, the neurotrophic properties of **1** and **2** were evaluated in detail in primary cultured fetal rat cortical neurons. A neurite outgrowth assay was performed using 18-day-old fetal rat cortical neurons in serum-free DMEM supplemented with B-27. Morphological evaluation, as shown in Fig. 3, indicated that mastigophorenes A (**1**) and B (**2**) not only promoted significant neurite outgrowth but also formed a network of neurons at 0.1 and 1 μM. A neuronal survival assay was carried out using the same neuronal cultures in serum-free DMEM supplemented with N-2, and the neuronal viability was assessed by the WST-8 assay. As summarized in Fig. 4, compounds **1** and **2** maintained neuronal survival at 0.1 and 1.0 μM, but lost their survival effect at 10 μM. These results suggest that mastigophorenes A (**1**) and B (**2**) can protect neurons from being insulted by toxic substances such as oxygen free radicals [32].

In addition, it should be noted that diasteroselective syntheses of mastigophorenes A (**1**) and B (**2**) with an atrop-enantioselective construction of the biaryl axis have been achieved by Bringmann [34], Meyers [35], and Feringa [36].

## Magnolol, honokiol and sesquiterpene-neolignans from *Magnolia* bark

**Fig. 5:**
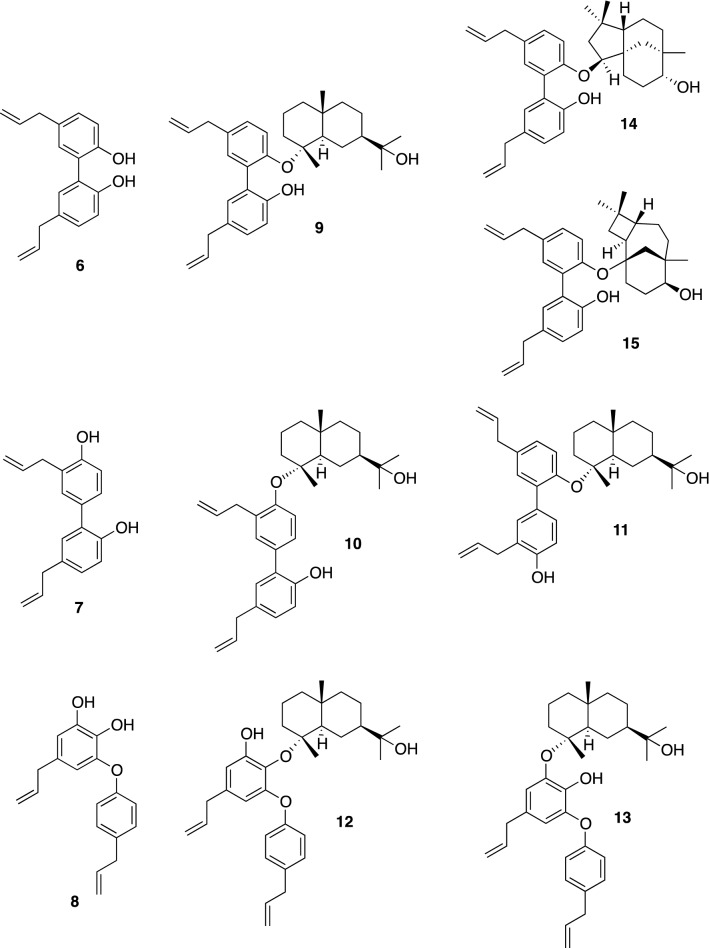
Neolignans **6**–**8** and sesquiterpene-neolignans **9**–**15** from *Magnolia obovata*

The bark of the *Magnolia* tree, *Magnolia obovata* Thumb. and *M. officinalis* Rhed. have been used in traditional herbal medicines in China, Korea and Japan. *Magnolia* bark is an important ingredient in Hange-kobokuto and Sai-boku-to preparations for the treatment of gastrointestinal disorders, anxiety and allergic diseases (Fig. 5). Moreover, other reported beneficial effects of *Magnolia* bark include anticancer, anti-inflammatory, antiplatelet and antioxidant activities [37, 38].

Magnolol (**6**) and honokiol (**7**), biphenyl neolignans, are the main constituents of *Magnolia* bark and have been reported to have a variety of biological properties such as antioxidative, antitumor, antidepressant, antidiabetic, anti-inflammatory, neuroprotective, and antimicrobial activities [39, 40]. In addition to their biological properties, magnolol (**6**) and honokiol (**7**) were found to have neurotrophic activity in primary cultured rat cortical neurons at concentrations ranging from 0.1 to 10 μM, but obovatol (**8**) had no activity even at 10 μM [41, 45]. Further studies on the minor components resulted in the isolation of various novel sesquiterpenes linked to biphenyl- or biphenylether-type neolignans termed sesquiterpene-neolignan, eudesmagnolol (**9**), eudeshonokiols A (**11**) and B (**10**), eudesobovatols A (**13**) and B (**12**), clovanemagnolol (**14**), and caryolanemagnolol (**15**) [42–44]. Among them, clovanemagnolol (**14**) and caryolanemagnolol (**15**) could not only accelerate neurite outgrowth but also activate choline acetyltransferase activity (ChAT) at the concentration of 0.01 μM [45].

**Scheme 2:**
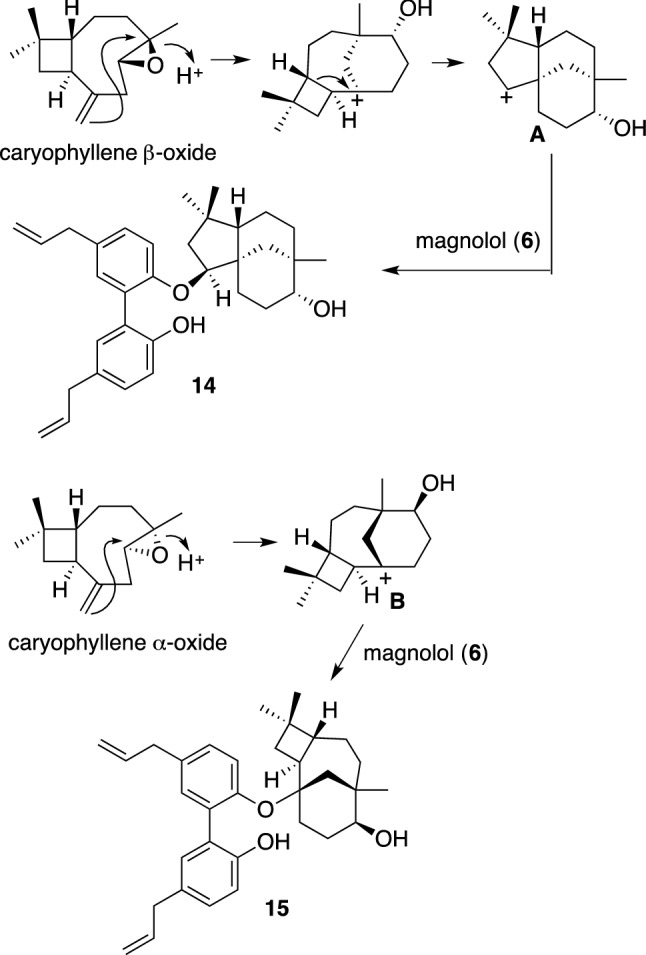
The proposed biosynthesis of clovanemagnolol (**14**) and caryolanemagnolol (**15**)

Clovanemagnolol (**14**) and caryolanemagnolol (**15**) are most likely to be converted from caryophyllene β-oxide and caryophyllene α-oxide, respectively, according to Barton’s results [45, 46]. The proposed biosynthetic pathway as shown in Scheme 2 is initiated by the oxidation of (–)-caryophyllene, providing caryophyllene β-oxide or caryophyllene α-oxide. Acidc activation of both epoxides leads to an intramolecular attack of the exocyclic alkene, generating the diastereomeric tricyclic cation intermediates **A** or **B**. Cation **A** is trapped by magnolol (**6**), directly forming clovanemagnolol (**14**), whereas cation **B** is trapped by **6**, giving rise to caryolanemagnolol (**15**). Siegel et al. synthesized **14** and **15** in two steps according to the postulated biosynthetic pathways [47, 49]. Synthesized **14** and **15** were confirmed to significantly promote neuronal growth at 0.01 μM in the primary cultured embryonic cortical neurons, similar to the neurotrophic effects of natural products [48, 49].

**Fig. 6:**
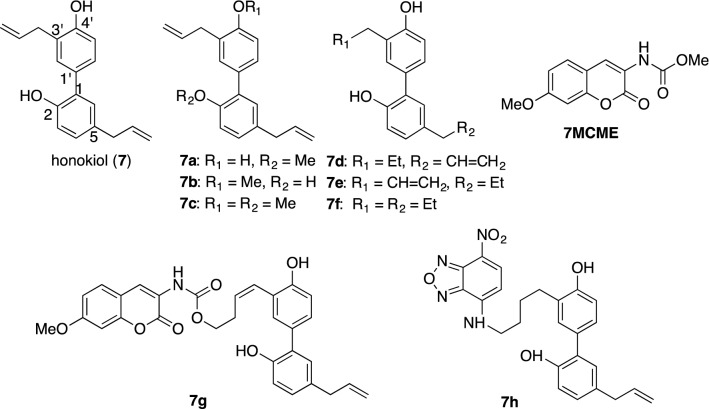
Structure of honokiol (**7**) and its analogs **7a**–**7f**, and florescent derivatives **7g**–**7h**

These results suggest that the lipophilic derivatives of simple biphenyl neolignans, magnolol (**6**) and honokiol (**7**) can enhance neurotrophic activity. Comparing the neurotrophic properties between **6** and **7**, **7** was found to be more potent than **6** [50]. For further developments of more effective derivative, honokiol (**7**) was synthesized in 21% yield over 14 steps by utilizing a Pd-catalyzed Suzuki–Miyaura reaction [51]. In addition, the structure–activity relationship between neurite outgrowth-promoting activity and the *O*-methylated and/or the hydrogenated analogs **7a**–**7f**, as summarized in Fig. 6, was examined in the primary cultured rat cortical neurons.

**Fig. 7:**
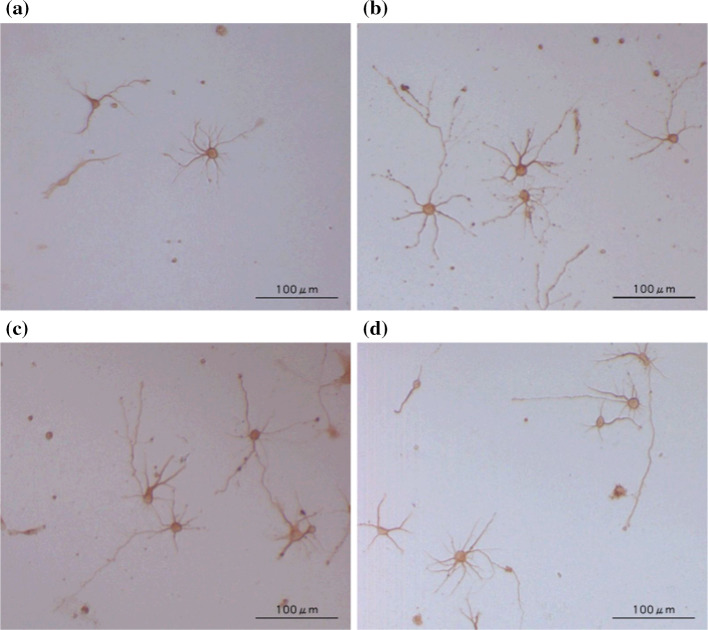
Morphology of cultured rat cortical neurons demonstrated with anti-MAP2 immunochemical staining. **a** Neurons in the presence of 0.5% ethanol as vehicle control; **b** neurons in the presence of 0.1μM **7**; **c** neurons in the presence of 1μM **7a**; **d** neurons in the presence of 0.1μM **7d**

**Fig. 8:**
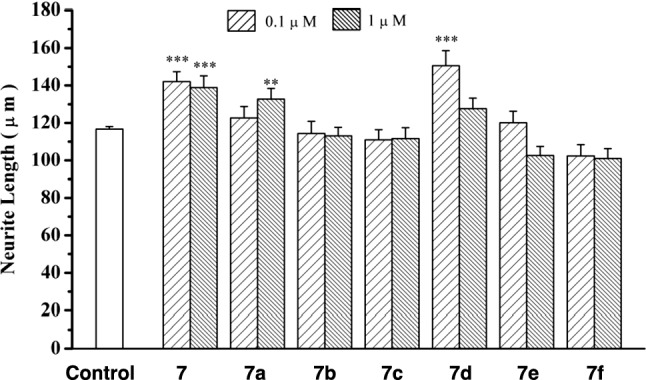
Quantitative analysis of anti-MAP2 immunochemically stained processes affected by honokiol (**7**) and its analogs **7a**–**7f**. In each group, the average lengths of the primary processes were determined from 100 neurons selected in random fields. **p < 0.01; ***p < 0.001 compared with control. Data presented here are derived from one of the two repeated experiments with similar results

As a result, honokiol (**7**), 2-*O*-methylhonokiol (**7a**), and 3′-dihydroallylhonokiol (**7d**) had striking effects on the morphology of cortical neurons as shown in Fig. 7. Analogs **7b**, **7c** and **7f** showed no enhancement of neurite extension. As shown in Fig. 8, quantitative analysis of the longest neurite length affected by each compound at the concentrations of 0.1 and 1 μM indicated that **7a** and **7d** have the potential to enhance neurite extension in cultured rat cortical neurons with a potency that is as high as that of **7**; whereas, analogs **7b**, **7c**, and **7f** showed diminished neurotrophic efficiency. Thus, these results suggest that the 4′-hydroxy group and the 5-allyl group are essential for honokiol-mediated neurite outgrowth-promoting activity (Figs. 7, 8). Based on the results of this SAR study, Gree and Chandrasekhar et al. synthesized 24 derivatives with various substituents at the 3′-allyl position of **7** and evaluated their neurotrophic effects in neurite outgrowth of differentiated Neuro2a cells after treatment with NGF but could not find new analogs that were more potent than **7** [52]. Recently, we successfully synthesized honokiol (**7**) in 74% yield over five steps [53], thereby a large amount of honokiol is now available for assessing its in vitro/in vivo biological activities and preparing a variety of derivatives.

**Fig. 9:**
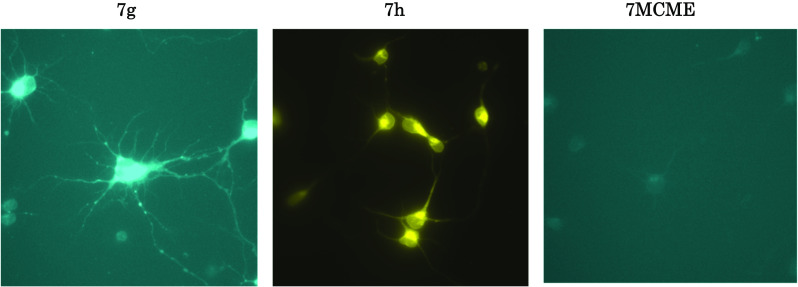
Distribution of fluorescent derivatives in primary cultured rat cortical neurons. Rat cortical neurons were incubated in the presence of 5-μM **7g**, **7h** and **7MCME** at 37 °C for 60 min followed by fluorescence imaging under microscope. Ex = 330 nm, Em = 431 nm for **7g** and **7MCME**. Ex = 470 nm, Em = 560 nm for 7 h

Next, after preparing several compounds and assessing their neurotrophic activity, we found two fluorescently labeled derivatives, 7-MCM (7-methoxycoumarin-3-carbonyl) (**7g**) and NBD (7-nitrobenzyl-2-oxa-1,3-diazolyl) (**7h**) that were suitable to be probe molecules to identify the intra/intercellular targets of **7**. Rat cortical neurons were incubated with 5 μM **7h**, **7g**, and **7MCME** for 1 h, and then their distributions in cortical neurons were monitored by fluorescence imaging under a microscope. As shown in Fig. 9, the apparent fluorescence was observed in intercellular regions, but the fluorescent molecule itself, **7MCME**, showed no fluorescence in any of the neurons. Taking a closer look at the fluorescent images, it is interesting to note the fluorescent vesicles assembled at the neck and/or branch of the dendrites in each cell body. These results suggest that honokiol could be taken up into cells and interact with specific targets, which would be associated with neurite outgrowth [54].

**Fig. 10:**
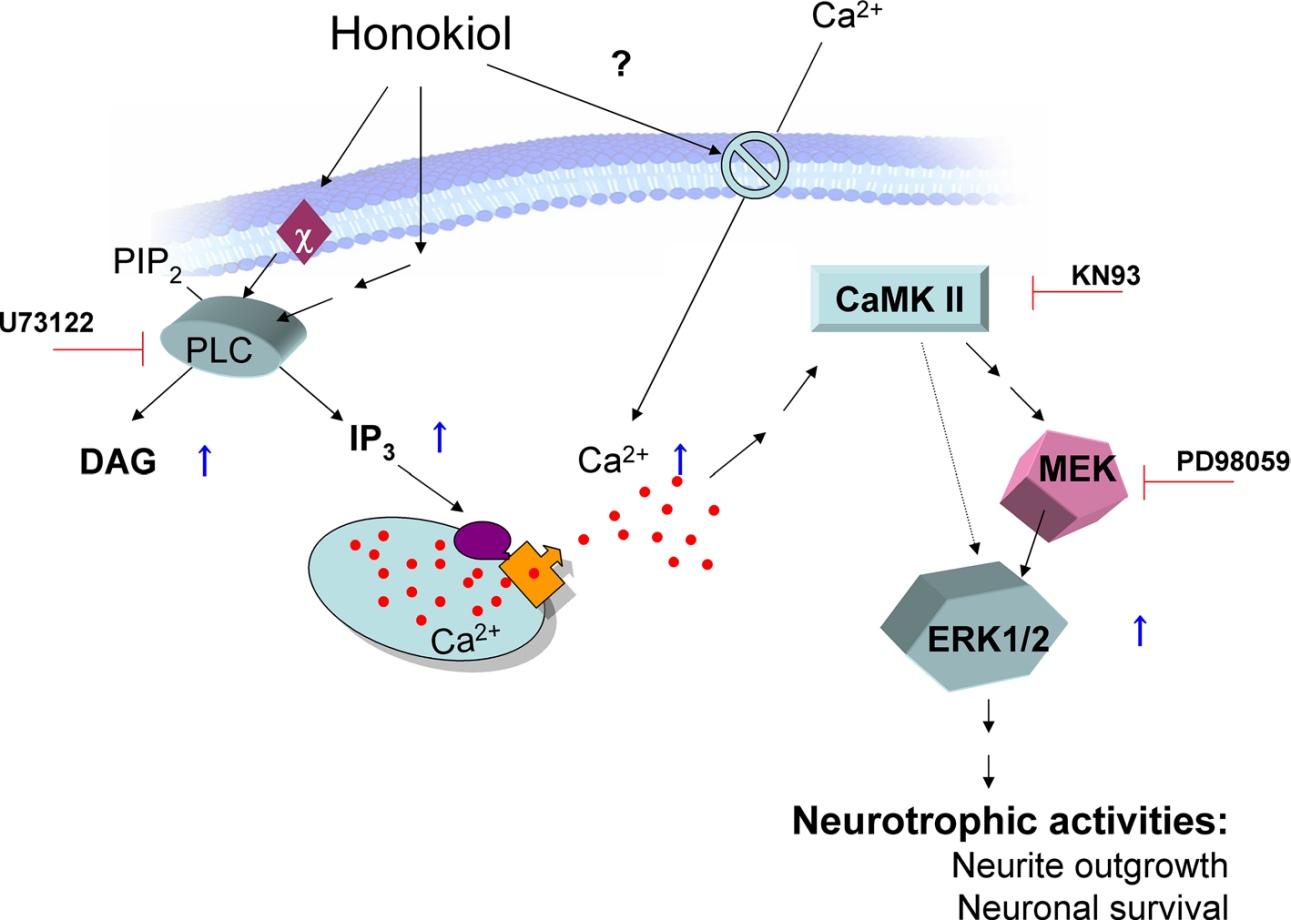
Proposed neurotrophic mechanism of honokiol (**7**) in cortical neurons. IP_3_ and DAG production, cytoplasmic free Ca^2+^ increase, and ERK1/2 phosphorylation are identified as effects of honokiol. The involvement of PLC, CaMK II, and MEK is shown using their specific inhibitor, U73122, KN93, and PD98059, respectively

Pharmacological studies of honokiol (**6**) and magnolol (**7**) have revealed that their effects on central nerves, such as depressant, muscle-relaxant, and anxiolytic effects, are mainly ascribed to their actions on GABA_A_ receptors [55]. In our search for neurotrophic compounds, **6** and **7** were identified as neurotrophic compounds that upregulate the activity of choline acetyltransferase in neuronal culture [45]. Furthermore, we found that **6** and **7** could promote neurite outgrowth and neuronal survival under serum-free conditions in cultured rat cortical neurons [50]. Additionally, it should be noted that magnolol could prevent the decrease in age-dependent neuronal loss in the hippocampus of senescence-accelerated mice (SAMP1) [56]. The intriguing effects of honokiol and magnolol prompted us to investigate the mechanisms underlying their neurotrophic actions using the cultured neurons. In general, neurotrophins such as NGF, BDNF, and NT-3 bind to the extracellular domain of the tyrosine kinase receptors TrkA, TrkB, and TrkC, respectively, and thereby activate the respective tyrosine kinase in the intracellular domain [57]. When target signal proteins bind to tyrosine kinases, they are phosphorylated to adopt active forms, and then transfer signals to their downstream. Among the NT-activated signaling molecules, Ca^2+^, MAPK (ERK), and Akt, are indispensable for transferring intracellular signals to nuclei [58]. First, we examined the intracellular Ca^2+^ response in primary cultured rat cortical neurons and human neuroblastoma SH-SY5Y cells by fluo-3 fluorescence imaging analysis. Magnolol and honokiol increased cytoplasmic free Ca^2+^ with a characteristic lag phase. The cytoplasmic free Ca^2+^ increase was independent of extracellular Ca^2+^ but dependent on the activation of phospholipase C and inositol 1,4,5-triphosphate (IP_3_) receptors, indicating an increase in cytoplasmic free Ca^2+^ through a phospholipase C-mediated pathway. Thus, **6** and **7** cause the release of Ca^2+^ from intracellular stores, resulting in an increase in cytoplasmic Ca^2+^ [59]. Regarding the effects of **6** and **7** on extracellular signal-regulated kinase (ERK1/2) and Akt, honokiol-induced neurite outgrowth in the cultured rat cortical neurons was significantly reduced by PD98059, a mitogen-activated protein kinase inhibitor, but not by LY294002, a phosphoinositide 3-kinase inhibitor. Honokiol also enhanced the phosphorylation of ERK1/2 in a dose-dependent manner; whereas, the effect of **7** on Akt phosphorylation was characterized by transient enhancement of 10 min and lasting inhibition after 30 min. The phosphorylation of ERK1/2 enhanced by **7** was inhibited by PD98059 as well as KN93, a Ca^2+^/calmodulin-dependent kinase II (CaMKII) inhibitor. Moreover, the products of the phosphoinositide-specific C (PLC)-derived inositol 1,4,5-triphosphate (IP_3_) and 1,2-diacylglycerol (DAG) were measured after honokiol treatment. Taking these results with our previous findings, as depicted in Fig. 10, signal transduction from PLC, IP_3_, Ca^2+^, and CaMKII to ERK1/2 has been proposed for a mechanism involved in honokiol-induced neurite outgrowth [60].

**Fig. 11:**
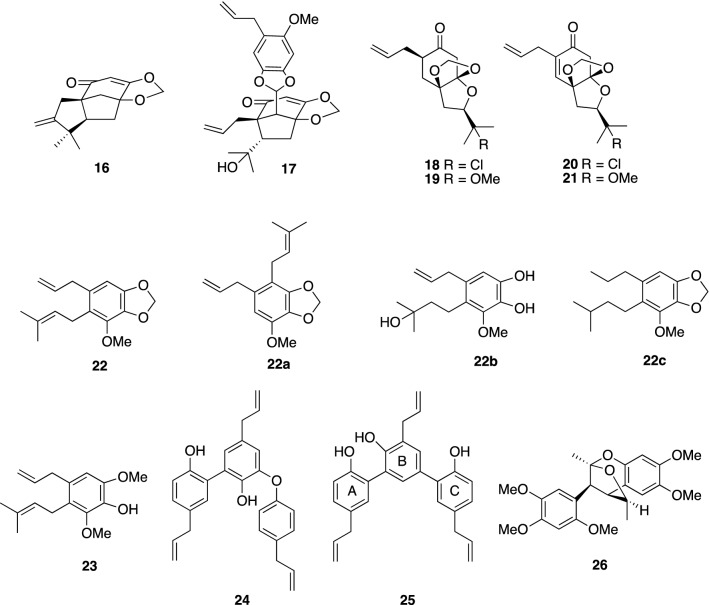
Prenylated C_6_–C_3_ compounds and neolignans from *Illicium* species

Furthermore, magnolol and honokiol were shown to be able to prevent age-related learning and memory impairment and cholinergic deficits in senescence-accelerated mice (SAMP8) [61]. Magnolol and honokiol were orally administered to 2-month-old SAMP8 mice once a day for 14 days. The SAMP8 mice showed significant impairment of learning and memory at 4 and 6 months of age. This age-related learning and memory impairment was prevented by pretreatment with either **6** (10 mg/kg) or **7** (1 mg/kg). In addition, **6** and **7** prevented age-related cholinergic defects and enhanced phosphorylation of Akt, a member of the prosurvival pathway, in the forebrain at 2 months of age (Fig. 11).

Recently, it was reported that **6** and **7** showed antidepressant-like effects on unpredictable chronic mild stress (UCMS)-treated rats by enhancing BDNF expression and serotonergic activity in the hippocampus [62, 63]. Matsui et al. reported that **6** significantly improved depressive behavior in olfactory bulbectomized (OBX) mice in the tail suspension test, significantly enhanced hippocampal neurogenesis and increased phosphorylation of Akt and cyclic AMP-responsive element-binding protein (CREB) [64]. These data demonstrate that magnolol (**6**) has antidepressant-like effects on behaviors and actions by enhancing hippocampal neurogenesis and neurotrophin-related intracellular signaling in mice.

Magnolol (**6**) and honokiol (**7**) are well known to have potent antioxidant effects [65]. Oral administration of **6** to C57BL/6N mice after 1-methyl-4-phenyl-1,2,3,6-terahydropyridinium (MPTP) treatment, an in vivo model of Parkinson’s model, almost completely prevented MPTP-induced lipid peroxidation in the stratum [66], suggesting that **6** has protective effects on the onset of cognitive impairments via an antioxidative mechanism. This is also consistent with the increasing lipid hydroperoxide level in the brain of SAMP8 at 2 months of age, which may be a cause of the age-related impairments and degeneration seen in the brain [67].

## Neurotrophic compounds from *Illicium* species

The genus *Illicium,* belonging to the family Illiciaceae, consists of approximately 40 species around the world. This genus is mainly distributed in the eastern North America, Mexico, the West Indies and eastern Asia. The fruits of the *Illicium* species are distinctive star-shaped follicles that emit a characteristic refreshing flavor. In particular, the fruits of *I. vernum* Hook, Chinese star anise, are the source of economically important product derived from this genus, which is widely used as a spice for flavoring food and beverages. Hence, essential oils have been the primary subject of chemical research on *Illicium* species, and the presence of volatile phenols has been reported as constituents of various parts of all *Illicium* species studied so far. The chemical constituents of the *Illicium* species are classified into three groups; sesquiterpenes, prenylated C_6_-C_3_ compounds, and triphenyl-neolignan (sesquineolignan). Some of these compounds are not only unique in their architectural structure but also exert intriguing bioactive effects on the neuronal system [68, 69].

**Scheme 3:**
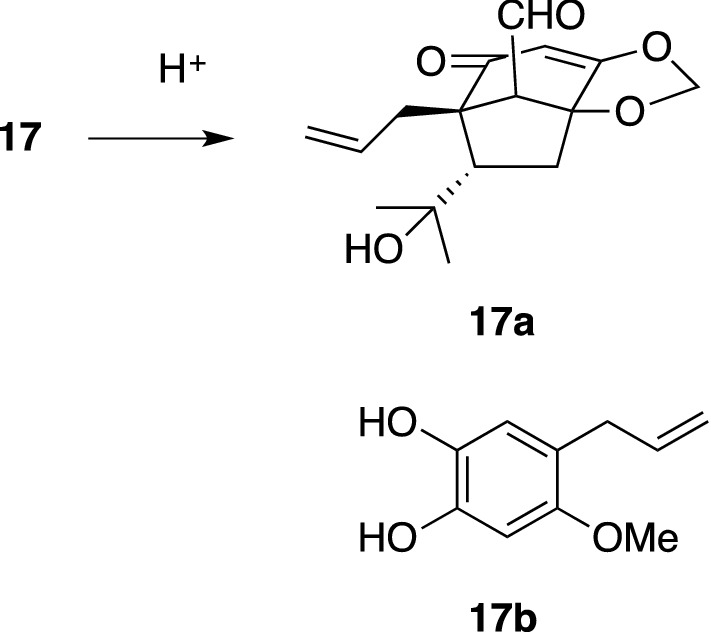
Acidic hydrolysis of **17**

We reported the isolation and structure of tricycloillicinone (**16**) [70] and (2*R*)-12-chloro-2,3-dihydroillicinone E (**18**) [71] from the woods of *I. tashiroi*. Compounds **16** and **18** were found to increase choline acetyltransferase (ChAT) activity by 143% and 228% at 30 μM in cultured P10 rat septal neurons, respectively. Lately, both compounds were shown to promote neurite outgrowth in NGF-mediated PC12 cells and primary cultured rat cortical neurons at concentrations as high as 50, 100 μM. On the other hand, compounds **19**–**21**, without a chlorine atom, did not have neurotrophic activity [72]. Another bicyclic nonaromatic phenylpropanoid, bicycloillicinone asarone acetal (**17**), was isolated from the same plant source as **16** and was found to enhance ChAT activity at 30 μM, which catalyzes the synthesis of acetylcholine from its precursor [73]. Acidic hydrolysis of **17** led to its core aldehyde, bicycloillicinone **17a**, and cathechol portion **17b** (Scheme 3).

The syntheses of **16** and the core structure **17a** were achieved by Danishefsky’s group [74]. Cholinesterase inhibitors such as donepezil and tacrine, which are capable of increasing neurotransmitter acetylcholine levels by inhibiting acethylcholinesterase (AChE) activity, are now in use for the treatment of AD [75]. Thus, compounds **16**–**18**, which can induce ChAT, an enzyme responsible for the biosynthesis of acetylcholine, should contribute to increased acetylcholine levels and support the survival of cholinergic neurons. As prenylated phenylpropanoids with neurotrophic activity, illicinin A (**22**) and compound **23** were isolated from *I. anisatum* [76]. Compounds **22** and **23** were found to significantly promote neurite outgrowth at concentrations ranging from 0.1 to 10 μM in primary cultured rat cortical neurons. Illicinin A (**22**) and its derivatives **22a**–**22c** were synthesized for structure–activity relationship studies by applying Pd-catalyzed Stille reactions and then were assessed for the neurite length of rat cortical neurons at 1 μM. As a result, compound **22c** showed reduced activity, whereas the others **22a** and **22b** showed comparative activity to illicinin A (**22**) or were more potent, thereby indicating that an allyl group in **22** is essential for exerting neurotrophic activity. In addition, the presence and position of the prenyl group in **22** were shown to play an important role in neurotrophic activity.

Typical neolignans, magnolol (**6**) and honokiol (**7**), were introduced in the early section of this review as having interesting neurotrophic properties. Macranthol (**25**) [77] and isodunnianol (**24**) [78], another neolignans bearing one phenyl group called sesquineolignan, showed neuroprotective activity at 5–10 μM [79] and neurite outgrowth-promoting activity at 0.1–10 μM in primary cultured rat cortical neurons [80] along with anti-AChE activity with an IC50 value of 13.0 μM [84]. In addition, macranthol (**25**) exerted an antidepressant-like activity in mice by increasing the expression of hippocampal BDNF [81], and the mechanism of its mediated antidepressant-like action was verified to be associated with BDNF-TrkB and downstream activation of the PI3K/Akt-Bcl-2/caspase-3 signal pathway [82]. Merrillianoid (**26**), a unique caged-shaped neolignan possessing a benzo-2,7-dioxa[3,2,1]octane moiety that was isolated as a racemic form from the leaves of *I. merrillianum*, influenced the NGF-induced neurite outgrowth of PC12 cells at concentrations from 1 to 10 μM, possibly by interacting with the TrkA receptor and downstream activation of ERK1/2 and MEK in the Ras/ERK signal cascade [83].

**Fig. 12:**
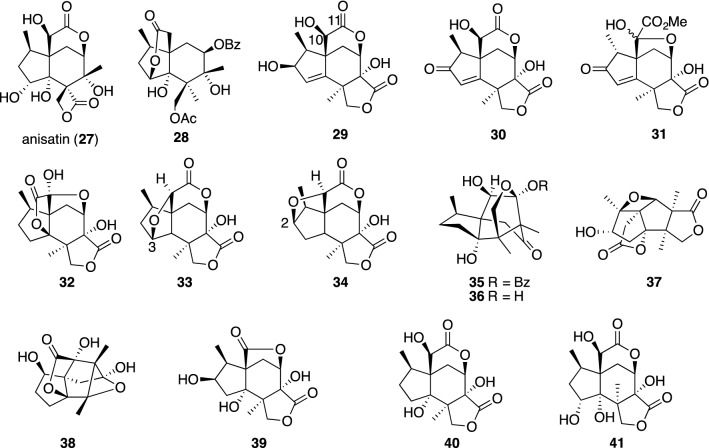
Anisatin (**27**) and *Illicium* sesquiterpenes having neurotrophic properties

The fruits of *I. anisatum* (Japanese star anise, “shikimi”) are known to be very toxic. Ingestion of these fruits causes convulsive symptoms, frequently leading to death. In 1952, Lane succeeded in the isolation of the pure toxic principle named anisatin (**27**), and its complete structure was later established by Yamada and Hirata [85]. Kawano et al., who continued to investigate the toxic substance in *I. anisatum*, succeeded in systematic studies on the chemical components in *Illicium* plants. Later, Schmidt and our group joined the chemical and biological studies of *Illicium* plants. A number of unique *seco*-prezizaane-type sesquiterpenes or so-called *Illicium* sesquiterpenes have been reported exclusively by the above three groups, and the occurrence of *Illicium* sesquiterpenes has been found to be limited to the genus *Illicium* [69]. Following the extensive chemical studies on *Illicium* sesquiterpenes, we turned our attention to neurotrophic properties but not neurotoxic activity for these sesquiterpenes. The isolation of sesquiterpenes from *Illicium* species, guided by the enhancement of ChAT activity, neurite outgrowth promotion and neuroprotective activity in primary cultured rat cortical neurons resulted in the discovery of a number of neurotrophic sesquiterpenes as shown in Fig. 12.

**Scheme 4:**
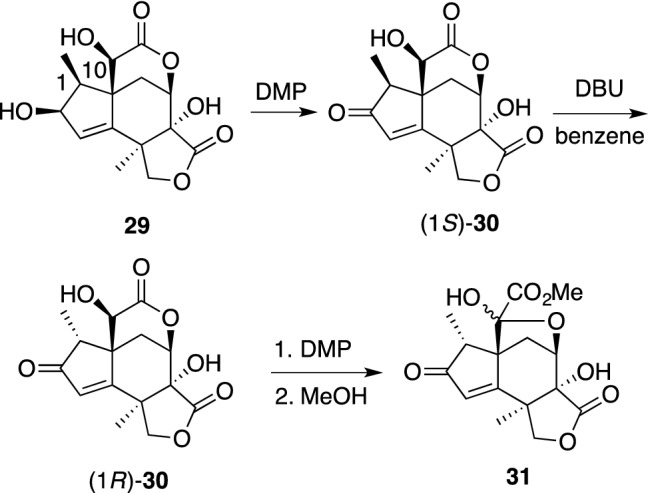
Conversion from **29** to **31**

Isodunnianin (**28**) was the first neurotrophic *Illicium* sesquiterpene isolated from the wood of *Illicium tashiroi* collected in Ishigaki Island, Japan, and it was shown to moderately enhance neurite-sprouting during the development of neurons and increase ChAT activity at 10 μM in primary fetal rat cortical neurons [86]. The structure of **28** was elucidated on the basis of the published NMR data of dunnianin [87], but later was corrected to **28** according to the revised structure of dunnianin [88]. Two majucin-type sesquiterpenes, (2*S*)-hydroxy-3,4-dehydroneomajucin (**29**) and jiadifenin (**31**), isolated from *Illicium jiadifengpi*, significantly promote the neurite outgrowth of primary cultured rat cortical neurons at concentrations ranging from 0.1 to 10 μM [89]. Jiadifenin (**31**) could be transformed from (2*S*)-hydroxy-3,4-dehydronemajucin (**29**) if the C-10 hydroxyl group is oxidized to a ketone. Thus, compound **30**, which was derived from **29** by DMP, was exposed to epimerization conditions at C-1 with DBU, leading to the thermodynamically more stable product (1*R*)-**30**. Next, DMP or Jones oxidation of (1*R*)-**30** [90] gave rise to jiadifenin (**31**) as an equilibrated mixture at the C-10 position in good yield, followed by the addition of MeOH to the reaction mixture (Scheme 4). This chemical transformation confirmed the absolute configuration of jiadifenin.

**Fig. 13:**
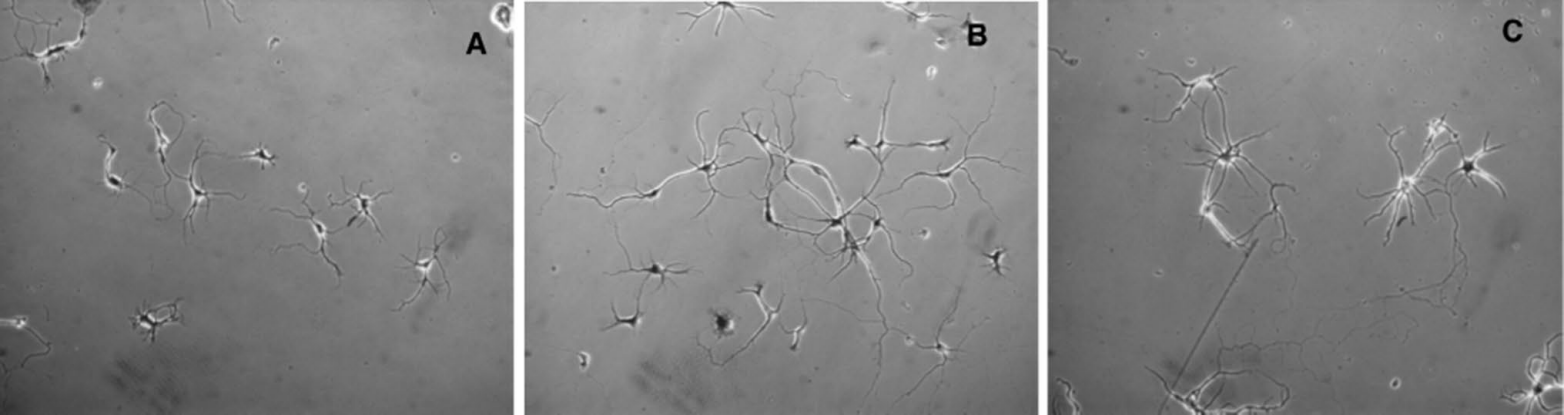
Neurite outgrowth-promoting activity of **32** in primary cultured rat cortical neurons. **a** Morphology of neurons in control groups, **b** morphology of neurons in 0.01 μM, **c** morphology of neurons in 10 μM

Another interesting majucin-subtype sesquiterpene, jiadifenolide (**32**), and jiadifenoxolanes A (**33**) and B (**34**), were isolated from the same plant [91]. Jiadifenolide (**32**) and jiadifenoxolane A (**33**) were found to significantly enhance the neurite outgrowth in primary cultured rat cortical neurons, in particular, jiadifenolide exhibited more potent activity at concentrations as low as 0.01 μM as shown in Fig. 13. These majucin-subtype sesquiterpenes were reported to promote neurite outgrowth of NGF-mediated PC12 cells but have no effect on PC12 cells in the absence of NGF [90]. Moreover, jiadifenolide (**32**) promoted neurite extension and significantly increased the total neurite area and length in neuronal cells derived from human induced pluripotent stem (iPS) cells at concentrations ranging from 1 to 10 μM [92].

**Fig. 14:**
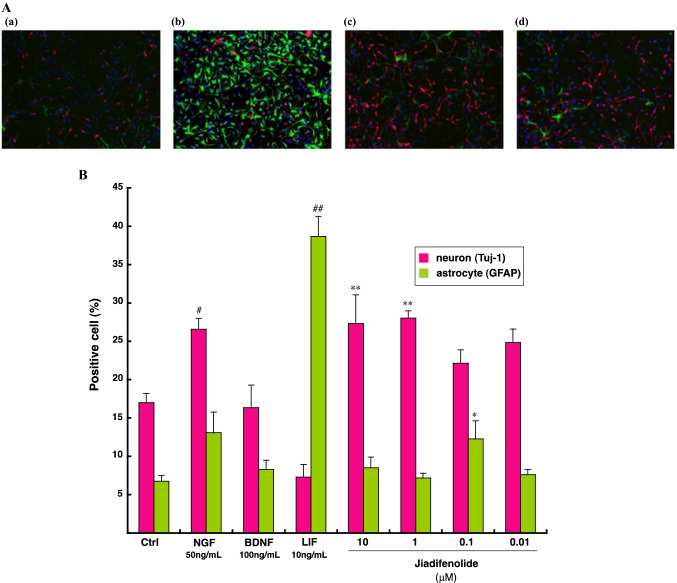
Effects of jiadifenolide on the neuronal differentiation of MEB5 cell line. **A** Morphological changes of MEB5 cells; blue, red, and green express nucleus, neuron, and astrocyte, respectively. a Control (0.5% EtOH), b leukemia inhibitory factor (LIF, 10 ng/mL), c nerve growth factor (NGF, 50 ng/mL), d jiadifenolide (10 μM). MEB5 cells were first cultured in the presence EGF at the density of 1.8 × 10^4^ cells/cm^2^, and then medium was changed to EGF-free medium. After 4 days in the absence of EGF, the cells were double labeled with antibodies to class III β-tubulin (Tuj-1, red) and glial fibrillary acidic protein (GFAP, green). **B** The percentage of the neuronal (red) and astrocytic (green) cells; the cells were maintained for 1 day in the proliferation medium, and then transferred to the differentiation medium containing vehicle (control) or jiadifenolide and cultured another 7 days. After immunostaining for Tuj-1 and GFAP, neuron, astrocyte, and total cell numbers were counted, and the ratio to total cells was calculated. Data were expressed as means ± SE (*n* = 5)

MEB5 is a multipotent stem cell line that can differentiate into neurons, astrocytes, and oligodendrocytes and, thus, is regarded as a potential tool to investigate compounds effective for the differentiation of CNS multipotent neuronal stem cells [22, 93, 94]. Furthermore, we attempted to assess the induction of differentiation of MEB5 cells by jiadifenolide. As shown in Fig. 14A, jiadifenolide significantly induced neuronal differentiation of MEB5 cells at 10 μM rather than astrocytic differentiation, with leukemina inhibitory factor (LIF) specifically induced as shown in Fig. 14A-b, B. The number of neurons at all the tested concentrations was greater in cultures treated with jiadifenolide than in control cultures (Fig. 14B). These results demonstrate that jiadifenolide promotes neuronal differentiation in the same manner as NGF.

**Scheme 5:**
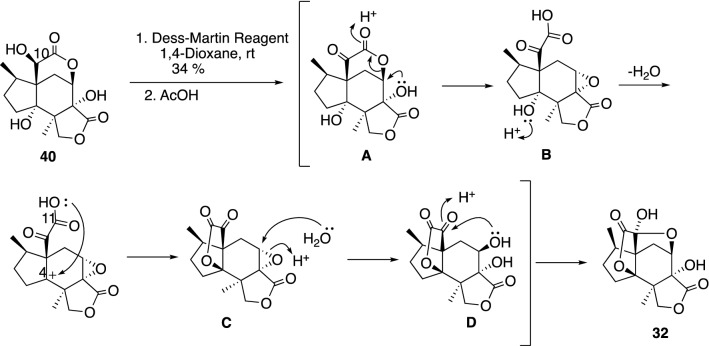
Direct oxidative conversion of **40** to **32**

Jiadifenolide (**32**) can be obtained straightforwardly from neomajucin (**40**) by DMP oxidation presumably through the proposed mechanism in Scheme 5 [91]. Jiadifenolide (**32**) and the other majucin-subtype sesquiterpenes **29**–**31** and **33** have attracted great attention from organic chemists due to their complex caged structural architecture and remarkable neurotrophic properties. Taking the structural similarity of the majucin-subtype sesquiterpenes into consideration, most synthetic strategies are divergent and comprehensive [18, 95–100].

**Scheme 6:**
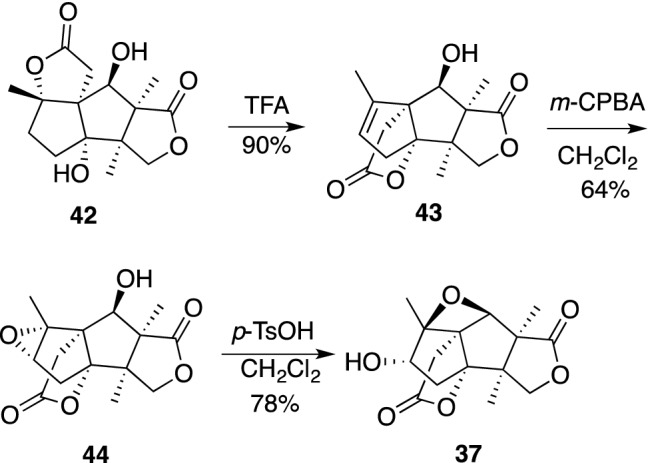
Partial synthesis of **37** from anislactone B (**42**)

Studies on *seco*-prezizaane-type sesquiterpenes from the *Illicium* species have culminated in their classification into further subgroups on the basis of a lactone type as follows: anisatin-subtype, pseudoanisatin-subtype, minwanensin-subtype, majucin-subtype, pseudomajucin-subtype, cycloparvifloralone-subtype, anislactone-subtype and *allo*-cedrane-subtype consisting of rare carbon skeletons. Merrilactone A (**37**), of the anislactone-subtype, from the pericarps of *Illicium merrillianum* was found to exhibit neurotrophic activity in primary cultured rat cortical neurons at a concentration as low as 0.1 μM [101]. The limited amount of **37** has prevented further biological studies. This encouraged us to attempt partial synthesis of merrilactone A (**37**) from anislactone B (**42**), which was available in a large quantity from the same plant [102]. First, a solution of **42** in trifluoroacetic acid (TFA) was refluxed to bring about the lactone transformation to the C-4 hydroxyl group with dehydration of the C-1 hydroxyl group, giving rise to **43** in good yield. Then, epoxidation of **43** with *m*-chloroperoxybenzoic acid (*m*-CPBA) proceeded in a highly stereoselective fashion to give **44**, which was treated with *p*-toluenesulfonic acid (*p*-TsOH) to produce merrilactone A (**37**) in 78% yield (Scheme 6). Following our reports, a number of excellent total syntheses of merrilactone A were published, and one should take a look at references [103, 104] for the details of each synthesis.

**Scheme 7:**
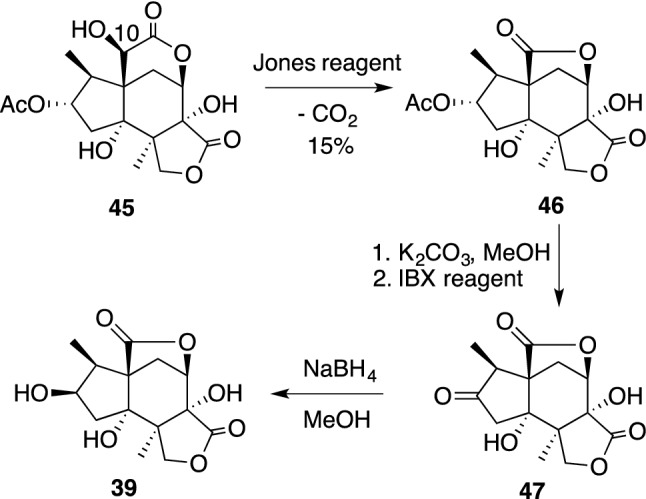
Chemical conversion from **45** to **39**

Tashironin (**35**) and 11-*O*-debenzoyltashironin (**36**) were isolated from *I. tashiroi* [105] and *I. merrillianum* [106]. Tashironin and its congeners [107] have a tricarbocyclic skeleton corresponding to the key intermediate, *allo*-cedrane in the biosynthesis of *seco*-prezizaane-type sesquiterpenes. Among *allo*-cedrane-type sesquiterpenes 11-*O*-debenzoyltashironin (**36**) exhibits solely neurotrophic features in primary cultured rat cortical neurons at 0.1–10 μM and the presence of a free acetal group at the C-11 position is essential for having neurotrophic activity [106]. In 2017, illisimonin A (**38**), which could be classified as a rare rearranged *allo*-cedrane, was isolated from the fruits of *I. simonsii* and was reported to show neuroprotective effects against oxygen–glucose deprivation-induced cell injury in SH-SY5Y cells with an EC50 value of 27.72 μM [108]. Recently, the absolute configuration of **38** was revised by its enantioselective synthesis [109]. The first example of a *seco*-prezizaane-type norsesquiterpene, (2*R*)-hydroxy-norneomajucin (**39**) was isolated from *I. jiadifengpi* and was added to a list of *Illicium* sesquiterpenes with neurotrophic activity [110]. The biosynthesis of **39** could be initiated by oxidation of the hydroxyl group at the C-10 position of neomajucin **40** to give a highly strained α-keto-δ-lactone, which would cause decarboxylation to lose one carbon, thereby leading to the less strained five-membered lactone **39**. As shown in Scheme 7, when 2-*O*-acetyl-(2*S*)-hydroxyneomajucin (**45**) was oxidized with Jones reagent, decarboxylation occurred spontaneously resulting in direct formation of the γ-lactone **46**, from which **39** was readily accessible after several reactions [110].

**Fig. 15:**
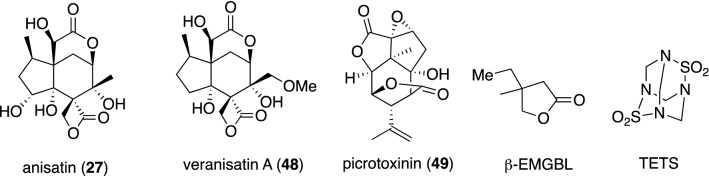
Convulsant sesquiterpenes and compounds that target primarily the GABA_A_ receptors

Neurotrophic sesquiterpenes **28**–**33** and **36**–**39** (Fig. 12) have significant structural homology with anisatin (**27**) and picrotoxinin (**49**) which cause convulsions but do not have neurotrophic properties. Picrotoxinin has been validated to elicit convulsion by binding to the GABA_A_ receptor and chloride anion blockade of the Cys-loop family of glutamate-gated chloride channels. Anisatin (**27**) and veranisatin (**48**) were also identified as picrotoxin-like, noncompetitive GABA-antagonists [111, 112]. Many of these convulsant terpenes contain γ-or δ-lactone motifs. Even the simple β-alkyl lactone β-EMGB exhibits convulsive activity by binding to the same site as picrotoxin. Based on structural homology with picrotoxinin (**49**) and anisatin (**27**) (Fig. 15), it has been postulated that neurotrophic *Illicium* sesquiterpenes would enhance neurite outgrowth by modulation of the Cys-loop family of GABA_A_ receptors, and a mechanistic link may exist between convulsant terpenes and neurotrophic *Illicium* sesquiterpenes [113].

In 2017, Shenvi et al. succeeded in the synthesis of (–)-11-*O*-debenzoyltashironin (**36**) and compared the effects of neurotrophic compounds **32** and **36**, and convulsive compounds **27** and **49** on primary cultures of rat cortical neurons in terms of the hyperexcitation expected from the antagonism of inhibitory channels. Although all four compounds similarly caused hyperexcitation of cortical neurons and inhibited GABA-evoked currents, the GABA antagonistic effects of **32** and **36** were tenfold weaker than those of **27** and **49**. Neurite outgrowth enhancement by **32** and **36** might be accounted for by a mechanism of chronic depolarization but more data are needed to validate this hypothesis in which convulsive and neurotrophic sesquiterpenes share a common target [114].

In 2018, Witkin et al. reported that jiadifenolide (**32**) and 11-*O*-debenzoyltashironin (**36**) did not cause convulsions in mice nor did they enhance or diminish convulsions induced by pentylenetetrazole (PTZ) although picrotoxinin (**49**) and tetramethylenedisulfotetramine (TETS) both induced convulsions. Furthermore, jiadifenolide and 11-*O*-debenzoyltashironin were verified to be less potent and less efficacious antagonists of GABA receptors than either picrotoxinin or TETS [115].

The underlying molecular mechanisms by which jiadifenolide (**32**) and its analogous *Illicium* sesquiterpenes exert their neurotrophic effects remain unknown despite the abovementioned hypothesis that they share a common target with the convulsant compounds including anisatin and picrotoxinin. We previously reported that jiadifenolide significantly promotes neurite outgrowth and cell growth as well as prevents death of neuronal precursor cells derived from human pluripotent stem cells (hiPSCs) [92]. By in silicon molecular network analysis of our comprehensive RNA sequencing results on **32**-treated human neuronal cells using KeyMolnet software, **32** was found to activate cellular communication network factor (CCN) signaling pathways by upregulating the mRNA expression of CCN2. In addition, the CCN2 protein was confirmed to exhibit neurotrophic effects and promote phosphorylation of the p44/42 MAPK protein in human neuronal cells. This result suggests that the molecular mechanism by which **32** exerts its neurotrophic effect is linked with CCN signaling [116]. It should be noted that this is the first discovery to connect neurotrophic effects with CCN signaling.

## Miscellaneous natural products with neurotrophic effects

**Fig. 16:**
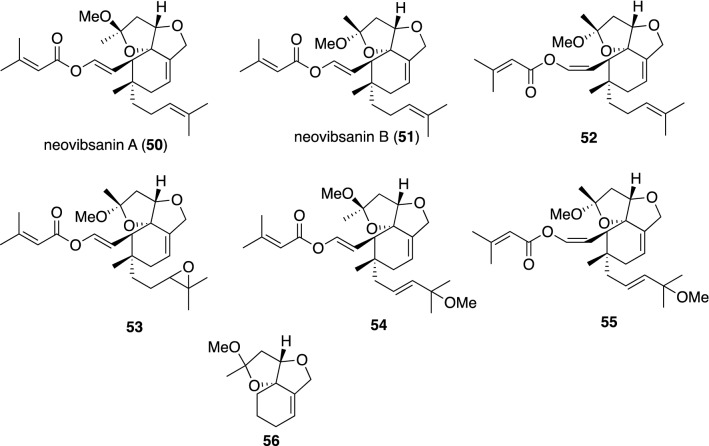
Neovibsanin-subtype diterpenes with neurotrophic effects

Vibsane-type diterpenoids rarely occur as natural products and have been limited to the isolation from *Viburnum* species thus far. The carbon skeletons of these diterpenes are further classified into three subtypes: those with an eleven-membered ring, those with a seven-membered ring, and the rearranged types (neovibsanins) [117, 118]. Among the three subtypes of vibsane-type diterpenoids, neovibsanins A (**50**) and B (**51**) and their congeners **52**–**55** with modifications on the prenyl group, as shown in Fig. 16, significantly enhanced the neurite outgrowth of NGF-mediated PC12 cells at concentrations ranging from 5 to 40 μM [119, 120]. A good synthetic achievement of neovibsanin B was accomplished by Imagawa et al. [121], who then extended their synthetic efforts to structure–activity relationship studies, resulting in elucidation of the minimum structure required for displaying neurite outgrowth activity in NGF-mediated PC12 cells (compound **56**) [122].

The Brazilian medicinal plant *Ptychopetalum olacoides* is known as a nerve tonic and is used for the treatment of chronic degenerative conditions of the central nervous system [123]. From the MeOH extract of this plant, a number of clerodane diterpenes **57**–**62** and **64**–**65** were isolated, among which ptychonal hemiacetal (**57**), ptychonal (**58**), 6α,7α-dihydroxyannonene (**61**), and 7α,20-dihydroxyannonene (**62**) exhibited NGF-potentiating activity in PC12 cells in the presence of NGF (20 ng/mL) at concentrations ranging from 0.1 to 40 μM [124, 125]. On the other hand, compounds **59**, **60**, and **63**–**65** had no effect on PC12 cells in the presence or absence of NGF. Compound **61** is the most potent NGF potentiator of these active compounds. The adjacent hydroxyl groups in **61** presumably contribute to its increased activity because the acetonide **63** loses activity at the same concentrations. In addition, the furan ring plays an important role in the appearance of NGF-potentiating activity since **64** and **65**, with modifications to the furan ring, showed no activity at all.

Four spirocyclic nortriterpenes, leonurusoleanolides A (**67**), B (**68**), C (**69**), and D (**70**) were isolated from the fruits of *Leonurus heterophyllus*. Compounds **67** and **68,** and compounds **69** and **70** exist as equilibrium mixtures of *E* and *Z* isomers. Mixtures of **67** and **68**, and **69** and **70**, significantly enhanced the neurite outgrowth of NGF-treated PC12 cells at concentrations ranging from 1 to 30 μM and also the parent compound, phlomistetraol B (**66**), possessed NGF-potentiating activity. However, **67**–**70** seemed to be more potent NGF potentiators than **66**, which had toxicity at concentrations higher than 30 μM [126].

**Fig. 17:**
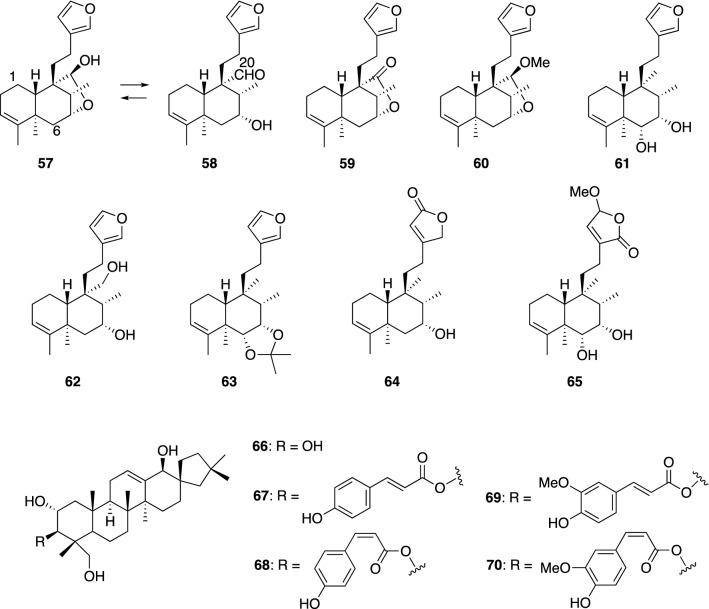
Other terpenes that enhance neurite outgrowth of NGF-mediated PC12 cells

**Fig. 18:**
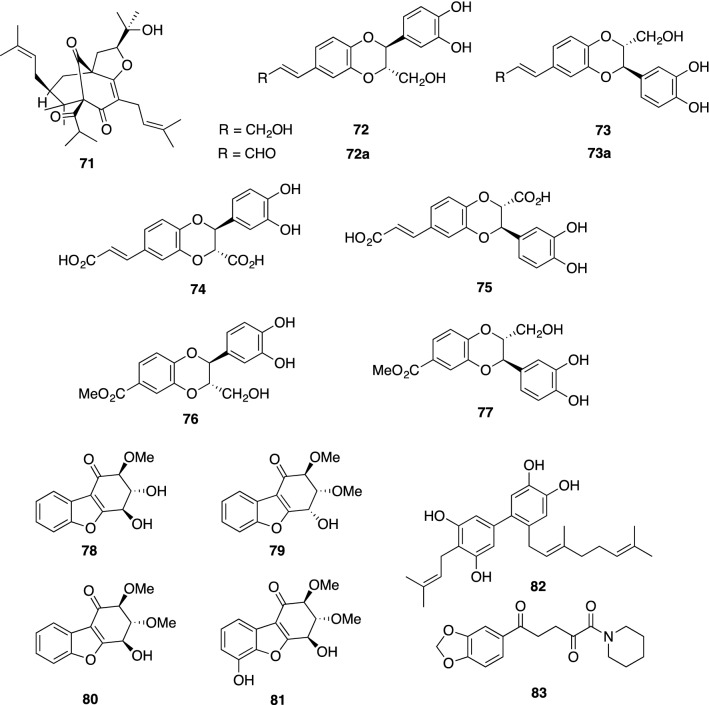
Garsubelline A (**71**) and aromatic compounds with neurotrophic effects

The polycyclic prenylated acylphloroglucinol (PPAP) family comprises a highly oxygenated and densely substituted bicyclo[3.3.1]nonane skeleton bearing prenyl or geranyl side chains that are rich in the family Clusiaceae (Guttiferae). *Hypericum perforatum*, commonly known as St. John’s wort, has attracted attention due to its antidepressant activity [127]. In the course of chemical studies on PPAPs of *Garcinia subelliptica* (Guttiferae) [128, 129], we found that garsubellin A (**71**) could increase the ChAT activity, a key enzyme for physiologic acetylcholine synthesis in the nervous system, by up to 154% at 10 μM in comparison with the control in primary cultured P10 rat septal neurons [130]. Owing to its important biological activity and architectural structure, garsubellin A (**71**) stimulated substantial synthetic effort, and the excellent synthetic achievements have been reported [127, 131–133]. More efficient synthetic methods of **71**, however, still have to be developed to supply sufficient quantities to explore its full biological characterization (Figs. 17, 18).

**Fig. 19:**
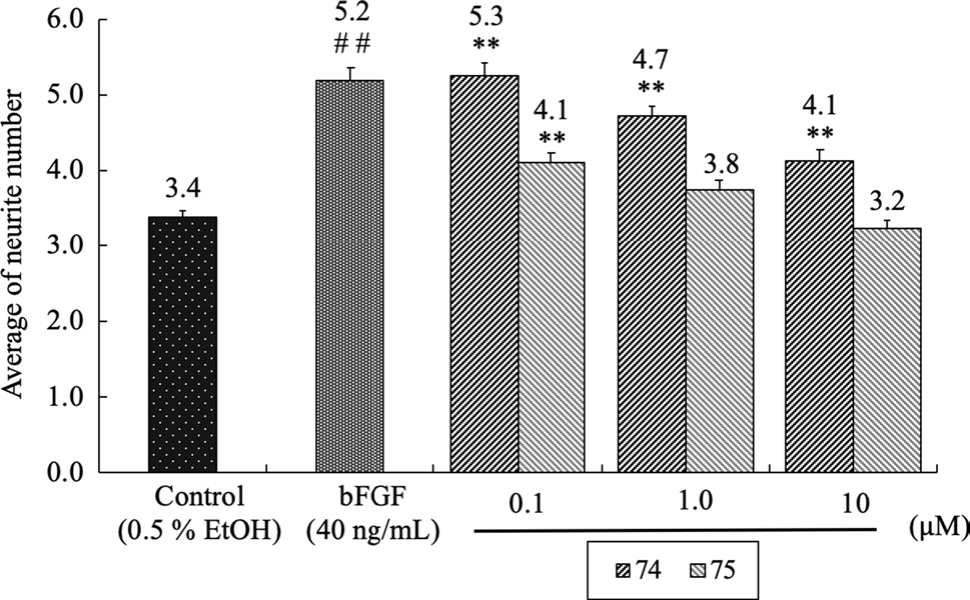
Increase of neurite number affected by **74** and **75**. The data are expressed ± S.E. (*n* = 80); student’s *t *test vs. control, ^##^*p* < 0.01; Dunnett’s *t *test vs. control, **p < 0.01 [138]

A unique group of the neolignans, such as americanol A (**72**), isoamericanol A (**73**), americanin A (**72a**), isoamericanin A (**73a**), americanoic acid A methyl ester (**76**) and isoamericanoic acid A methyl ester (**77**), which are characterized by having a 1,4-benzodioxane ring and have diverse and significant biological activities, occur exclusively in the seeds of *Phytolacca americana* L. (Phytolaccaceae) [134, 135]. In particular, americanol A and isoamericanol A were found to enhance not only ChAT activity but also neurite outgrowth at 10 μM in primary cultured fetal rat hemispheres [136]. Neolignans **72** and **73** would be formed by oxidative dimerization of the corresponding monomeric unit, coniferyl alcohol. In fact, caffeic acid was subjected to horseradish peroxidase (HRP)-catalyzed oxidative conditions to give rise to dicarboxylic acids **74** and **75**, which were converted to **72** and **73**, respectively, followed by sequential reductions [137]. We reexamined the neurotrophic activities of **72**–**77** in primary cultured rat cortical neurons. In addition to **72** and **73**, americanoic acid A methyl ester (**76**) was found to exhibit potent neurite outgrowth activity at 0.1 μM; whereas, the activities of compounds **74**, **75** and **77** were comparable with control cultures. Although compounds **74** and **75** had no effects on neurite outgrowth, they induced significant neuritogenesis such as increasing the number of neurite branches in the concentration range from 0.1 to 10 μM in a similar manner to basic fibroblast growth factor (bFGF) as shown in Fig. 19 [138].

Novel polyoxygenated benzofuran derivatives, namely, ribisin A (**78**), ribisin B (**79**), ribisin C (**80**), and ribisin D (**81**), were isolated from the fruiting bodies of *Phellinus ribis*, which is used in the East Asian countries as a traditional medicine for enhancing immunity and gastrointestinal cancer. Beznofurans **78**–**81** showed marked neurite outgrowth-promoting activity in NGF-mediated PC12 cells at concentrations ranging from 1 to 30 μM; whereas, none of these four compounds had morphological effects on PC12 cells in the absence of NGF. Their absolute configurations on the chiral positions were elucidated by applying the CD exciton chirality method to the readily derived *p*-bromobenzoate [139]. Chemoenzymatic total syntheses of **78–81** were achieved by two groups [140, 141], and therefore, the absolute stereochemistry of ribisin C (**80**) was revised as its *ent*-form.

A prenylated and geranylated biphenyl derivative, clusiparalicoline A (**82**), was isolated from the roots of *Clusia paralicola* and showed modest cytotoxicity in the KB cell line [142]. We achieved the first synthesis of **82** by applying sequential palladium-catalyzed Stille and Suzuki reactions, and synthetic **82** was assessed by our neuronal cell assay. As a result, **82** was found to exhibit potent neurite outgrowth-promoting activity at concentrations from 0.1 to 1.0 μM in primary cultured rat cortical neurons, but the application of a higher dose than 10 μM induced the death in all neurons [143]. Although **82** shows cytotoxicity at high concentrations, the biaryl moiety of **82** may have some positive effects on the development and survival of neurons in the same manner as honokiol and magnolol, which have a specific affinity for neuronal cells [45].

Piperine (1-piperoylpiperidine), a pungent nitrogenous substance, is a main alkaloid in various piper species. These piper plants are commonly used as household spices as well as important traditional medicine in many Asian countries. With regard to piperine on cognitive function, recent pharmacological studies have shown that piperine possesses cognitive-enhancing activity [144] and improves both memory impairment and neurodegeneration in rats with cognitive defects induced by the ethylcholine aziridinium ion (AF64A) [145]. These reports prompted us to evaluate the neurotrophic properties of the fruits of the Javanese long pepper, *Piper retrofractum*, which are called Cabe Jawa and are used as one of the ingredients in the Indonesian natural medicine jamu. The methanolic extract of *P. retrofractum* fruits exhibited neurite outgrowth-promoting activity in NGF-mediated PC12 cells. Bioassay-guided fractionation resulted in the isolation of the active component, piperodione (**83**), which is the first example of piperine oxidized at the C-2 and C-5 positions. Compound **83** showed potent NGF-potentiating activity in PC12 cells at concentrations ranging from 0.1 to 10 μM, but piperine had no effect on PC12 cells at the same dose as **83** [146]. Our study has demonstrated that piperine is not the active constituent responsible for the observed neurotrophic activity of *P. retrofractum* fruits. Recently, the total synthesis of piperodione (**83**) was reported from two groups, and **83** was verified to have NGF-potentiating activity but not cytotoxicity at all [147, 148]. Now, with large quantities of **83** in hand, further pharmacological studies of **83** are in progress.

Finally, it should be noted that a comprehensive review on (–)-talaumidin, a neurotrophic 2,5-biaryl-3,4-dimethylhydrofuran lignan, has been published quite recently [149]. Given the increasing interest in neurotrophin-mimic compounds, we suggest that readers refer to a recent review covering another topic of neurotrophic natural products.

## Conclusions

In this review, we have introduced neurotrophin-mimicking natural products discovered by our group and have focused on the chemical and biological features of neurotrophic compounds but have not emphasized their synthetic achievements, because there are already some excellent reviews on this subject [18, 104, 150] that summarize the overview of their organic syntheses. Endogenous neurotrophins (NGF, BDNF etc.) are a class of polypeptidyl agents that promote neurogenesis, neuron survival, process outgrowth, and synaptic connectivity in the development of the neuronal system and neuronal plasticity in adult neurons. As mentioned in the introduction, the application of neurotrophins to treat neurodegenerative diseases suffers serious drawbacks in practice due to their unfavorable properties. Consequently, a small molecule non-peptidyl agent that mimics the functions of neurotrophic factors would be an attractive alternative for the treatment and/or protection of neurodegenerative diseases. This idea prompted us to search for plant-derived small molecules that have neurotrophic effects on three neuronal cells: PC12 cells, primary cultured rat cortical neurons, and MEB5 neuronal stem cells. Beginning in 1988, structurally unique isocuparane dimers, mastigophorenes A (**1**) and (**2**) were discovered as neurotrophic compounds by our group. Since then, a number of compounds with neurotrophic activity have been discovered from various plants. However, many active compounds have not gained access to detailed biological investigations, in particular, to in vivo animal models and their underlying neurotrophic mechanisms remain obscure because insufficient quantities of material are an often-encountered problem associated with natural products. To overcome this issue, many groups have been engaged in the synthesis of neurotrophic natural products. Jiadifenolide (**32**) is a good example. Chemical synthesis has supplied a sufficient quantity of **32** to undertake detailed biological studies, which leads to the possibility of clinical utility for jiadifenolide.

Through this review, we hope that organic chemists and medicinal chemists will share their interests and efforts with neurotrophin-mimicking small molecules, which are expected to not only play a critical role in chemical control over each step in the neural circuit life model (neurogenesis, differentiation, neurite outgrowth, death), as proposed in Fig. 1, but also make a positive contribution to protecting cognitive impairment in a superaged society.

## References

[1] Data from “Welfare White Paper 2018 Edition”. www.mhlw.go.jp/wp/haksusyo/kousei/16/backdata/01-01-01-02.html

[2] Thompson LM (2008). Neurodegeneration: a question of balance. Nature.

[3] Data from “Dementia Policy Promotion Comprehensive (New Orange Plane) 2018. www.mhlw.go.jp/stf/seisakunitsuite/bunya/0000076236.html

[4] Querfurth HW, Lafera FM (2010). Alzheimer’s disease. N Engl J Med.

[5] Mattson MP (2004). Pathways towards and away from Alzheimer’s disease. Nature.

[6] Hardy J, Selkoe DJ (2002). The amyloid hypothesis of Alzheimer’s disease: progress and problems on the road to therapeutics. Science.

[7] Reardon S (2015). Alzheimer’s drugs show progress. Nat Digest.

[8] Lang AE (2010). Clinical trials of disease-modifying therapies for neurodegenerative diseases: the challenge and the future. Nat Med.

[9] Xu X, Warrington AE, Bieber AJ, Rodriguez M (2012). Enhancing central nervous system repair-the challenges. CNS Drugs.

[10] Levitan IB, Kaczmarek LK (2002). The neuron: cell and molecular biology.

[11] Gibon J, Barker PA (2017). Neurotrophins and proneurotrophins: focus on synaptic activity and plasticity in the brain. Neuroscientist.

[12] Huang E (2003). TRK receptors: roles in neuronal signal transduction. Annu Rev Biochem.

[13] Dechant G, Barde Y-A (2002). The neurotrophin receptor p75^NTR^: novel functions and implications for disease of the nervous system. Nat Neurosci.

[14] Hefti F (1998). Nerve growth factor treatment for Alzheimer’s diseases: the experience of the first attempt at intracerebral neurotrophic factor therapy. Handb Exp Pharmacol.

[15] Tuszynski MH, Thal L, Pay M, Salmon DP, Sang UH, Bakay R, Patel P, Blesch A, Vahlsing HL, Ho G, Tong G, Potkin SG, Fallon J, Hansen L, Mufson EJ, Kordower JH, Gall C, Conner J (2005). A phase 1 clinical trial of nerve growth factor gene therapy for Alzheimer disease. Nat Med.

[16] Nagahara AH, Tuszynski MH (2011). Potential therapeutic uses of BDNF in neurological and psychiatric disorders. Nat Rev Drug Discov.

[17] Swain C, Harper S, Pollack S, Smith R, Hefti F (1998). Neurotrophic factor mimetics. Handb Exp Pharmacol.

[18] Xu J, Lacoske MH, Theodorakis EA (2014). Neurotrophic natural products: chemistry and biology. Angew Chem Int Ed.

[19] Kubo M (2015). Search for neurotrophin-mimic natural products for prevention and treatment of neurodegenerative disease. Yakugaku Zasshi.

[20] Greene LA, Tischler AS (1976). Establishment of a noradrenergic clonal line of rat adrenal pheochromocytoma cells which respond to nerve growth factor. PNAS.

[21] Abe K, Takayanagi M, Saito H (1990). Effects of recombinant human basic fibroblast growth factor and its modified protein CS23 on survival of primary cultured neurons from various regions of fetal rat brain. Jpn J Pharmacol.

[22] Nakagaito Y, Satoh M, Kuno H, Iwama T, Takeuchi M, Hakura A, Yoshida T (1998). Establishment of an epidermal growth factor-dependent, multipotent neural precursor cell line. In Vitro Cell Dev Biol Animal.

[23] Fukuyama Y, Kodama M (1996). Search for novel neurotrophic factor-like substances in natural products. FFI J.

[24] Li Y, Ohizumi Y (2004). Search for constituents with neurotrophic factor-potentiating activity from the medicinal plants of Paraguay and Thailand. Yakugaku Zasshi.

[25] Akagi M, Matsui N, Akae H, Hirashima N, Fukuishi N, Fukuyama Y, Akagi R (2015). Nonpeptide neurotrophic agents useful in the treatment neurodegenerative diseases such as Alzheimer’s diseases. J Pharmacol Sci.

[26] Asakawa Y, Ludwiczuk A, Nagashima F, Toyota M, Hashimoto T, Tori M, Fukuyama Y, Harinantenaina L (2009). Bryophytes: bio- and chemical diversity, bioactivity and chemosystematics. Heterocycles.

[27] Fukuyama Y, Toyota M, Asakawa Y (1988). Mastigophorenes: novel dimeric isocuparane-type sesquiterpenoids from the liverwort *Mastigophora diclados*. J Chem Soc Chem Commun.

[28] Fukuyama Y, Asakawa Y (1991). Novel neurotrophic isocuparane-type sesquiterpene dimers, mastigophorenes A, B, C and D, isolated from the liverwort *Mastigophora diclados*. J Chem Soc Perkin Trans.

[29] Matsuo A, Yuki S, Nakayama M (1986). Structures of *ent*-herbertane sesquiterpenoids displaying antifungal properties from the liverwort *Herberta adunca*. J Chem Soc Perkin Trans.

[30] Asou H, Iwasaki N, Hirano S, Dahl D (1985). Mitotic neuroblasts in dissociated cell cultures from embryonic rat cerebral hemispheres express neurofilament protein. Brain Res.

[31] Bringmann G, Pabst T, Rycroft DS, Connolly JD (1999). First synthesis of mastigophorenes A and B, by biomimetic oxidative coupling of herbertenediol. Tetrahedron Lett.

[32] Fukuyama Y, Matsumoto K, Tonoi Y, Yokoyama R, Takahashi H, Minami H, Okazaki H, Mitsumoto Y (2001). Total syntheses of neuroprotective mastigophorenes A and B. Tetrahedron.

[33] Fukuyama Y, Kiriyama Y, Kodama M (1996). Total synthesis of herbertenediol, an isocuparane sesquiterpene isolated from liverworts. Tetrahedron Lett.

[34] Bringmann G, Pabst T, Henschel P, Kraus J, Peters K, Peters E-M, Rycroft DS, Connolly JD (2000). Nondynamic and dynamic kinetic resolution of lactones with stereogentic centers and axes: stereoselective total synthesis of herbertendiol and mastigophorenes A and B. J Am Chem Soc.

[35] Degnan AP, Meyers AI (1999). Total syntheses of (-)-herbertenediol, (-)-mastigophorene A, and (-)-mastigophorene B. Combined utility of chiral bicyclic lactams and chiral aryl oxazolines. J Am Chem Soc.

[36] Buter J, Heijinen D, Vila C, Hornillos V, Otten E, Giannerini M, Minnaard AJ, Feringa BL (2016). Palladium-catalyzed, *tert*-butyllithium-mediated dimerization of aryl halides and its application in the atropselective total synthesis of mastigophorene A. Angew Chem Int Ed.

[37] Lee YJ, Lee YM, Lee CK, Jung JK, Han SB, Hong JT (2011). Therapeutic applications of compounds in the Magnolia family. Pharmacol Ther.

[38] Guerra-Araiza C, Alvarez-Mejia AL, Sanchez-Torres S, Farfan-Garcia E, Mondragon-Lozano R, Pinto-Almazan R, Salgado-Ceballos H (2013). Effect of natural exogenous antioxidants on aging and on neurodegenerative diseases. Free Radic Res.

[39] Yang C, Zhi X, Xu H (2016). Advances on semisynthesis. Total synthesis, and structure-activity relationships of honokiol and magnolol derivatives. Mini Rev Med Chem.

[40] Sarrica A, Kirika N, Romeo M, Salmona M, Diomede L (2018). Safety and toxicology of magnolol and honokiol. Planta Med.

[41] Fukuyama Y, Nakade K, Minoshima Y, Yokoyama R, Zhai H, Mitsumoto Y (2002). Neurotrophic activity of honokiol on the cultures of fetal rat cortical neurons. Bioorg Med Chem Lett.

[42] Fukuyama Y, Otoshi Y, Kodama M (1989). Novel neurotrophic sesquiterpene-neolignans from *Magnolia obovata*. Tetrahedron Lett.

[43] Fukuyama Y, Otoshi Y, Kodama M (1990). Structure of clovanemagnolol, a novel neurotrophic sesquiterpene-neolignan from *Magnolia ovovata*. Tetrahedron Lett.

[44] Fukuyama Y, Otoshi Y, Nakamura K, Kodama M, Sugawara M, Nagasawa M (1990). Structure of eudesmagnolol and eudeshonokiol, novel sesquiteprene-neolignans isolated from *Magnolia obovata*. Chem Lett.

[45] Fukuyama Y, Otoshi Y, Miyoshi K, Nakamura K, Kodama M, Nagasawa M, Hasegawa T, Okazaki H, Sugawara M (1992). Neurotrophic sesquiterpene-neoligans from *Magnolia obovata*: structure and neurotrophic activity. Tetrahedron.

[46] Aebi A, Barton DHR, Lindsey AS (1953) Sesquiterpenpoids. Part III. The stereochemistry of caryophyllene. J Chem Soc (C) 3124–3129

[47] Cheng X, Harzdorf NL, Shaw T, Siegel D (2010). Biomimetic syntheses of the neurotrophic natural products caryolanemagnolol and clovanemagnolol. Org Lett.

[48] Khaing Z, Kang D, Camelio AM, Schmidt CE, Siegel D (2011). Hippocampal and cortical neuronal growth mediated by the small molecule natural product clovanemagnolol. Bioorg Med Chem Lett.

[49] Cheng X, Harzdorf N, Khaing Z, Kang D, Camelio AM, Shaw T, Schmidt CE, Siegel D (2012). Neuronal growth promoting sesquiterpene-neolignans; syntheses and biological studies. Org Biomol Chem.

[50] Fukuyama Y, Nakada K, Minoshima Y, Yokoyama R, Zhai H, Mitusmoto Y (2002). Neurotrophic activity of honokiol on the cultures of fetal rat cortical neurons. Bioorg Med Chem Lett.

[51] Esumi T, Makado G, Zhai H, Shimizu Y, Mitsumoto Y, Fukuyama Y (2004). Efficient synthesis and structure–activity relationship of honokiol, a neurotrophic biphenyl–type neolignan. Bioorg Med Chem Lett.

[52] Kumar VP, Reddy RG, Vo DD, Chakravarty S, Chandrasekhar S, Gree R (2012). Synthesis and neurite growth evaluation of new analogues of honokiol, a neolignan with potent neurotrophic activity. Bioorg Med Chem Lett.

[53] Harada K, Arioka C, Miyakita A, Kubo M, Fukuyama Y (2014). Efficient synthesis of neurotrophic honokiol using Szuzuki-Miyaura reactions. Tetrahedron Lett.

[54] Zhai H, Nakade K, Mitsumoto Y, Fukuyama Y (2003). Neurotrophic activity and mechanism of honokiol and magnolol. Yakugaku Zasshi.

[55] Ai J, Wang X, Nielsen M (2001). Honokiol and Magnolol selectively interact with GABA_A_ receptor subtypes in vitro. Pharmacology.

[56] Matsui N, Nakashima H, Ushio Y, Tada T, Shirono A, Fukuyama Y, Nakade K, Zhai H, Yasui Y, Fukuishi N, Akagi R, Alagi M (2005). Neurotrophic effect of magnolol in the hippocampal CA1 region of senescence-accelerated mice (SAMP1). Biol Pharm Bull.

[57] Levitan IB, Kaczmatek LK (2002). The neuron, cell and molecular biology.

[58] Pollack SJ, Harper SJ (2002). Small molecule Trk receptor agonists and other neurotrophic factor mimetics. Curr Drug Targets CNS Neural Disord.

[59] Zhai H, Nakade K, Mitsumoto Y, Fukuyama Y (2003). Honokiol and magnolol induce Ca^2+^ mobilization in rat cortical neurons and human neuroblastoma SH-SY5Y cells. Eur J Pharmacol.

[60] Zhai H, Nakade K, Oda M, Mitsumoto Y, Akagi M, Sakurai J, Fukuyama Y (2005). Honokiol-induced neurite outgrowth promotion depends on activation of extracellular signal-regulated kinases (ERK1/2). Eur J Pharmacol.

[61] Matsui N, Takahashi K, Takeichi M, Kuroshita T, Noguchi K, Yamazaki K, Tagashira H, Tsutsui K, Okada H, Kido Y, Yasui Y, Fukuishi N, Fukuyama Y, Akagi M (2009). Magnolol and honokiol prevent learning and memory impairment and cholinergic deficit in SAMP8 mice. Brain Res.

[62] Li LF, Lu J, Li XM, Xu CL, Deng JM, Qu R, Ma SP (2012). Antidepressant-like effect of magnolol on BDNF up-regulation and serotonergic system activity in unpredictable chronic mild stress treated rats. Phytother Res.

[63] Wang C, Gan D, Wu J, Liao M, Liao X, Ai W (2018). Honokiol exerts antidepressant effects in rats exposed to chronic unpredictable mild stress by regulating brain derived neurotrophic factor level and hypothalamus-pituitary-adrenal axis activity. Neurochem Res.

[64] Matsui N, Akae H, Hirashima N, Kido Y, Tanabe S, Koseki M, Fukuyama Y, Akagi M (2016). Magnolol eanhances hippocampal neurogenesis and exerts antidepressant-like effects in olfactory bulbectomized mice. Phytother Res.

[65] Haraguchi H, Ishikawa H, Shirataki N, Fukuda A (1997). Antiperoxidative activity of neolignans from *Magnolia obovata*. J Pharm Pharmacol.

[66] Muroyama A, Fujita A, Lv C, Kobayashi S, Fukuyama Y, Mitsumoto Y (2012). Magnolol protects against MPTP/MPP^+^-induced toxicity via inhibition of oxidative stress in *in vitro* and in vivo models of Parkinson’s disease. Parkinson’s Dis.

[67] Yasui F, Ishibashi M, Matsugo S, Kojo S, Oomura Y, Sasaki K (2003). Brain lipid hydroperoxide level increases in senescence-accelerated mice at an early age. Neurosci Lett.

[68] Fukuyama Y, Huang J-M, Jordal MM (2004). Chemical constituents of the genus *Illicium*. Illicium, Pimpinella and Foeniculum.

[69] Fukuyama Y, Huang J-M (2005) Chemistry and neurotrophic activity of *seco*-prezizaane- and anislatone-type sesquiterpenes from *Illicium* species. In: Atta-ur-Rahman (ed) Studies in natural products chemistry, Bioactive natural products (part L), vol 32. Elsevier, Amsterdam, pp 395–428

[70] Fukuyama Y, Shida N, Kodama M, Chaki H, Yugami T (1995). Tricycloillicinone, a novel prenylated C_6_–C_3_ compound increasing choline acetyltransferase (ChAT) activity, isolated from *Illicium tashiroi*. Chem Pharm Bull.

[71] Fukuyama Y, Okamoto K, Kubo M, Shida N, Kodama M (1994). New chlorine-containing prenylated C_6_–C_3_ compounds increasing choline acetyltransferase (ChAT) activity in culture of postnatal rat septal neurons from *Illicium tashiroi*. Chem Pharm Bull.

[72] Fukuyama Y, Shida N, Hata Y, Anzaki S, Kodama M (1994). Prenylated C_6_–C_3_ compounds related to illicinone E from *Illicium tashiroi*. Phytochem.

[73] Fukuyama Y, Hata Y, Kodama M (1997). Bicycloillicinone asarone acetal: a novel prenylated C_6_–C_3_ compound increasing choline acetyltransferase (ChAT) activity from *Illicium tashiroi*. Planta Med.

[74] Pettus TRR, Inoue M, Chen X-T, Danishefsky SJ (2000). A fully synthetic route to the neurotrophic illicinones: syntheses of tricycloillicinone and bicycloillicinone aldehyde. J Am Chem Soc.

[75] Giacobini E (1998). Cholinesterase inhibitors for Alzheimer’s disease therapy: from tacrine to further applications. Neurochem Int.

[76] Takaoka S, Takaoka N, Minoshima Y, Huang J-M, Kubo M, Harada K, Hioki H, Fukuyama Y (2009). Isolation, synthesis, and neurite outgrowth-promoting activity of illicinin A from the flowers of *Illicium anisatum*. Tetrahedron.

[77] Kouno I, Hashimoto A, Kawano N, Yang C-S (1989). New sesqui-neolignan from the pericarps of *Illicium macranthum*. Chem Pharm Bull.

[78] Kouno I, Morisaki T, Hara Y, Yang C-S (1991). Two new sesquineolignans from the bark of *Illicium dunnianum*. Chem Pharm Bull.

[79] Moriyama M, Huang J-M, Yang C-S, Hioki H, Kubo M, Harada K, Fukuyama Y (2007). Structure and neurotrophic activity of novel sesqui-neolignans from the pericarps of *Illicum fargessi*. Tetrahedron.

[80] Moriyama M, Huang J-M, Yang C-S, Kubo M, Harada K, Hioki H, Fukuyama Y (2008). Two new sesquiterpenoids and two new prenylated phenylpropanoids from *Illicium fargesii*, and neuroprotective activity of macranthol. Chem Pharm Bull.

[81] Li J, Geng D, Xu J, Weng LJ, Liu Q, Yi LT (2013). Antidepressant-like effect of macranthol isolated from *Illicium dunnianum* tutch in mice. Eur J Pharmacol.

[82] Luo L, Liu XL, Li J, Mu RH, Liu Q, Yi LT, Geng D (2015). Macranthol promotes hippocampal neuronal proliferation in mice via BDNF-TrkB—PI3K/Akt signaling pathway. Eur J Pharmacol.

[83] Tian X, Yue R, Zeng H, Li H, Shan L, He W, Shen Y, Zhang W (2015). Distinctive effect on nerve growth factor-induced PC12 cell neurite outgrowth by two unique neolignan enantiomers from *Illicium merrillianum*. Sci Rep.

[84] Dong C-F, Liu L, Luo H-R, Li X-N, Guan Z-Y, Wang Y-F (2012). Sesquilignans and sesquiterpenoid from the stem barks of *Illicium simonsii* and their anti-AChE activity. Nat Prod Bioprospect.

[85] Takada S, Nakamura S, Yamada K, Hirata Y (1966). Isolation and structure of neoanisatin. Tetrahedron Lett.

[86] Fukuyama Y, Shida N, Kodama M (1993). Isodunnianin: a new sesquiterpene enhancing neurite outgrowth in primary culture of fetal rat cerebral hemisphere from *Illicium tashiroi*. Planta Med.

[87] Kouno I, Kawano N, Yang C-S (1988). New pseudoanisatin-like sesquiterpene lactones from the bark of *Illicium dunnianum*. J Chem Soc Perkin Trans.

[88] Schmidt TJ, Schmidt HM, Müller E, Peters W, Fronczek FR, Truesdale A, Fischer NH (1998). New sesquirepene lactones from *Illicium floridanum*. J Nat Prod.

[89] Yokoyama R, Huang J-M, Yang C-S, Fukuyama Y (2002). New *seco*-prezizaane-type sesquiterpenes, jiadifenin with neurotrophic activity and 1,2-dehydroneomajucin from *Illicium jiadifengpi*. J Nat Prod.

[90] Carcache DA, Cho YS, Hua Z, Tian Y, Li Y-M, Danishefsky SJ (2006). Total synthesis of (±)-jiadifenin and studies directed to understanding its SAR: probing mechanistic and stereochemical issues in palladium-mediated allylation of enolate-like structures. J Am Chem Soc.

[91] Kubo M, Okada C, Huang J-M, Harada K, Hioki H, Fukuyama Y (2009). Novel pentacyclic *seco*-prezizaane-type sesquiterpenoids with neurotrophic properties from *Illicium jiadifengpi*. Org Lett.

[92] Shoji M, Nishioka M, Minato H, Harada K, Kubo M, Fukuyama Y, Kuzuhara T (2016). Neurotrophic activity of jiadifenolide on neuronal precursor cells derived from human induced pluripotent stem cells. Biochem Biophy Res Commun.

[93] Satoh M, Sugino H, Yoshida T (2000). Activin promotes astrocytic differentiation of a multipotent neural stem cell line and an astrocyte progenitor cell line from murine central nervous system. Neurosci Lett.

[94] Nakagaito Y, Yoshida T, Satoh M, Takeuchi M (1995). Effects of leukemia inhibitory factor on the differentiation of astrocyte progenitor cells from embryonic mouse cerebral hemispheres. Dev Brain Res.

[95] Trzoss L, Xu J, Lacoske MH, Mobley WC, Theodorakis EA (2013). *Illicium* sesquiterpenes: divergent synthetic strategy and neurotrophic activity studies. Chem Eur J.

[96] Harada K, Imai A, Uto K, Carter RG, Kubo M, Hioki H, Fukuyama Y (2015). Synthesis of jiadifenin using Mizoroki-Heck and Tuji-Trost reactions. Tetrahedron.

[97] Lu H-H, Martinez MD, Shenvi RA (2015). An eight-step gram-scale synthesis of (–)-jadifenoilde. Nat Chem.

[98] Shen Y, Li L, Pan Z, Wang Y, Li J, Wang K, Wang X, Zhang Y, Hu T, Zhang Y (2015). Protecting-group-free total synthesis of (–)-jiadifenolide: development of [4 + 1] annulation toward multisubstituted tetrahydrofurans. Org Lett.

[99] Cheng X, Micalizio GC (2016). Synthesis of neurotrophic *seco*-prezizaane sesquiterpenes (1*R*, 10*S*)-2-oxo-3,4-dehydroneomajucin, (2*S*)-hydroxy-3,4-dehydroneomajucin, and (–)-jiadifenin. J Am Chem Soc.

[100] Hung K, Condakes ML, Novaes LFT, Harwood SJ, Morikawa T, Yang Z, Maimone TJ (2019). Development of a terpene Feedstock-based oxidative synthetic approach to the *Illicium* sesquiterpenes. J Am Chem Soc.

[101] Huang J-M, Yokoyama R, Yang C-S, Fukuyama Y (2000). Merrilactone A, a novel neurotrophic sesquiterpene dilactone from *Illicium merrillianum*. Tetrahedron Lett.

[102] Huang J-M, Yang C-S, Tanaka M, Fukuyama Y (2001). Structures of merrilactones B and C, novel anislactone-type sesquiterpenes from *Illicium merrillianum,* and chemical conversion of anislactone B to merrilactone A. Tetrahedron.

[103] Urabe D, Inoue M (2009). Total syntheses of sesquiterpenes from *Illicium* species. Tetrahedron.

[104] Greaney MF, Shi L, Nazef N, Harmata M (2013). Total synthesis of (±)-anislactone A and (±)-merrilactone A. Strategies and tactics in organic synthesis.

[105] Fukuyama Y, Shida N, Kodama M (1995). Tashironin, a plausible biosynthetic precursor of anisatin-type sesquiterpenes. Tetrahedron Lett.

[106] Huang J-M, Yokoyama R, Yang C-S, Fukuyama Y (2001). Structure and neurotrophic activity of *seco*-prezizaane-type sesquiterepenes from *Illicium merrillianum*. J Nat Prod.

[107] Song W-Y, Ma Y-B, Bai X, Zhang X-M, Gu Q, Zheng Y-T, Zhou J, Chen J-J (2007). Two new compounds and anti-HIV active constituents from *Illicium verum*. Planta Med.

[108] Ma S-G, Li M, Lin M-B, Li L, Liu Y-B, Qu J, Li Y, Wang X-J, Wang R-B, Xu S, Hou Q, Yu S-S (2017). Illisimonin A, a caged sesquiterpenoid with tricyclo[5.2.1.0^1,6^]decane skeleton from the fruits of *Illicium simonsii*. Org Lett.

[109] Burns AS, Rychnovsky SD (2019). Total synthesis and structure revision of (–)-illisimonin A, a neuroprotective sesquiterpenoid from the fruits of *Illicium simonsii*. J Am Chem Soc.

[110] Kubo M, Kobayashi K, Huang J-M, Harada K, Fukuyama Y (2012). The first examples of *seco*-prezizaane-type norsesquiterpenoids with neurotrophic activity from *Illicium jiadifengpi*. Tetrahedon Lett.

[111] Kudo Y, Oka J, Yamada K (1981). Anisatin, a potent GABA antagonist, isolated from *Illicium anisatum*. Neurosci Lett.

[112] Nakamura T, Okuyama E, Yamazaki M (1996). Neurotropic components from star anise (*Illcium verum* Hook, fil.). Chem Pharm Bull.

[113] Shevi RA (2016). Neurite outgrowth enhancement by jiadifenolide: possible target. Nat Prod Rep.

[114] Ohtawa M, Krambis MJ, Cerne R, Schkeryantz JM, Witkin JM (2017). Synthesis of (–)-11-*O*-debenzoyltashronin: neurotrophic sesquiterpenes cause hyperexcitation. J Am Chem Soc.

[115] Witkin JM, Shenvi RA, Li X, Gleason SD, Weiss J, Morrow D, Gatow JT, Wakulchik M, Ohtawa M, Lu H-H, Martinez MD, Schkeryantz JM, Carpenter TS, Lightstone FC, Cerne R (2018). Pharmacological characterization of the neurotrophic sesquiterpene jiadifenolide reveals a non-convulsant signature and potential for progression in neurodegenerative diseases studies. Biochem Pharmcol.

[116] Shoji M, Ueda M, Nishioka M, Minato H, Seki M, Harada K, Kubo M, Fukuyama Y, Suzuki Y, Aoyama E, Takigawa M, Kuzuhara T (2019). Jiadifenolide induces the expression of cellular communication network factor (CCN) genes, and CCN2 exhibits neurotrophic activity in neuronal precursor cells derived from human induced pluripotent stem cells. Biochem Biophy Res Commun.

[117] Fukuyama Y, Kubo M, Esumi T, Harada K, Hioki H (2010). Chemistry and biological activities of vibsane-type diterpenoids. Heterocycles.

[118] Miwa K, Esumi T, Imagawa H, Fukuyama Y (2014) Chemical diversity of vibsane-type diterpenoids and neurotrophic activity and synthesis of neovibsanin. In: Atta-ur-Rahman (ed) Studies in natural products chemistry, vol 43. Elsevier, Amsterdam, pp 41–78

[119] Kubo M, Kishimoto Y, Harada K, Hioki H, Fukuyama Y (2010). NGF-potentiating vibsane-type diterpenoids from *Viburnum sieboldii*. Bioorg Med Chem Lett.

[120] Kubo M, Nakai M, Harada K, Fukuyama Y (2019). Structure of seven new vibsane-type diterpenoids from *Viburnum awabuki*. Tetrahedron.

[121] Imagawa H, Saijo H, Kurisaki T, Yamamoto H, Kubo M, Fukuyama Y, Nishizawa M (2009). Total synthesis of (±)-neovibsanin B. Org Lett.

[122] Imagawa H, Saijo H, Yamaguchi H, Maekawa K, Kurisaki T, Yamamoto H, Nishizawa M, Oda M, Kabura M, Nagahama M, Sakurai J, Kubo M, Nakai M, Makino K, Ogata M, Takahashi H, Fukuyama Y (2012). Syntheses of structurally-simplified and fluorescently-labeled neovibsanin derivatives and analysis of their neurite outgrowth in PC12 cells. Bioorg Med Chem Lett.

[123] Hashimoto G (1996). Illustrated encyclopedia of Brazilian medicinal plants.

[124] Tang W, Hioki H, Harada K, Kubo M, Fukuyama Y (2008). Clerodane diterpenoids with NGF-potentiating activity from *Ptychopetalum olacoides*. J Nat Prod.

[125] Tang W, Kubo M, Harada K, Hioki H, Fukuyama Y (2009). Novel NGF-potentiating diterpenoids from a Brazilian medicinal plant, *Ptychopetalum olacoides*. Bioorg Med Chem Lett.

[126] Liu Y, Kubo M, Fukuyama Y (2012). Spirocyclic nortriterpenoids with NGF-potentiating activity from the fruits of *Leonurus heterophyllus*. J Nat Prod.

[127] Ciochina R, Grossman RB (2006). Polycyclic polyprenylated acylphloroglucinols. Chem Rev.

[128] Fukuyama Y, Kaneshi A, Tani N, Kodama M (1993). Subellinone, a polyisoprenylated phloroglucinol derivative from *Garcinia subelliptica*. Phytochemistry.

[129] Fukuyama Y, Minami H, Kuwayama A (1998). Garsubellins, polyisoprenylated phloroglucinol derivatives from *Garcinia subelliptica*. Phytochemistry.

[130] Fukuyama Y, Kuwayama A, Minami H (1997). Garsubellin A, a novel polyprenylated phloroglucin derivative, increasing choline acetyltransferase (ChAT) activity in postnatal rat septal neuron cultures. Chem Pharm Bull.

[131] Ahmad NA, Rodeschini V, Simpkins NS, Ward SE, Blake AJ (2007). Synthesis of polyprenylated acylphloroglucinols using bridgehead lithiation: the total synthesis of racemic clusianone and a formal synthesis of racemic garsubellin A. J Org Chem.

[132] Uwamori M, Nakada M (2013). Stereoselective total synthesis of garsubellin A. J Antibiot.

[133] Shen X, Ting CP, Xu G, Maimone TJ (2020). Programmable meroterpene synthesis. Nat Commun.

[134] Woo WS, Kang SS, Seligmann O, Chari VM, Wagner H (1980). The structure of new lignans from the seeds of *Phytolacca americana*. Tetrahedron Lett.

[135] Hasegawa T, Fukuyama Y, Koshino K, Nakagawa K, Tori M, Asakawa Y (1987). Structure of isoamericanin A, a prostaglandin I_2_ inducer, isolated from the seeds of *Phytolacca americana* L. Chem Lett.

[136] Fukuyama Y, Hasegawa T, Toda M, Kodama M, Okazaki H (1992). Structures of americanol A and isoamericanol A having a neurotrophic property from the seeds of *Phytolacca americana*. Chem Pharm Bull.

[137] Matsumoto K, Takahashi H, Miyake Y, Fukuyama Y (1999). Convenient syntheses of neurotrophic americanol A and isoamericanol A by HRP catalyzed oxidative coupling of caffeic acid. Tetrahedron Lett.

[138] Takahashi H, Yanagi K, Ueda M, Nakade K, Fukuyama Y (2003). Structures of 1,4-benzodioxane derivatives from the seeds of *Phytolacca americana* and their neuritogenic activity in primary cultured rat cortical neurons. Chem Pharm Bull.

[139] Liu Y, Kubo M, Fukuyama Y (2012). Nerve growth factor-potentiating benzofuran derivatives from the medicinal fungus *Phellinus ribis*. J Nat Prod.

[140] Lan P, Banwell MG, Willis AC (2014). Chemoenzymatic total syntheses of ribisins A, B, and D, polyoxygenated benzofuran derivatives displaying NGF-potentiating properties. J Org Chem.

[141] Boyd DR, Sharma ND, McGivern CJ, Stevenson PJ, Hoering P, Allen CCR (2019). Chemoenzymatic synthesis of (–)-ribisins A and B from dibenzo[*b*,*d*]furan. J Org Chem.

[142] Seo E-K, Huang L, Wall ME, Wani MC, Navarro H, Mukherjee R, Farnsworth NR, Kinghorn AD (1999). New biphenyl compounds with DNA strand-scission activity from *Clusia paralicola*. J Nat Prod.

[143] Takaoka S, Nakade K, Fukuyama Y (2002). The first total synthesis and neurotrophic activity of clusiparalicoline A, a prenylated and geranylated biaryl from *Clusia paralicola*. Tetrahedron Lett.

[144] Wattanathorn J, Chonpathompikunlert P, Muchimapura S, Piprem A, Tankamnerdthai O (2008). Piperine, the potential functional food for mood and cognitive disorders. Food Chem Toxicol.

[145] Chonpathompikunlert P, Wattanathorn J, Muchimapura S (2010). Piperine, the main alkaloid of Thai black pepper, protects against neurodegeneration and cognitive impairment in animal model of cognitive deficit like condition of Alzheimer’s disease. Food Chem Toxicol.

[146] Kubo M, Ishii R, Ishino Y, Harada K, Matsui N, Akagai M, Kato E, Hosoda S, Fukuyama Y (2013). Evaluation of constituents of *Piper retrofractum* Fruits on neurotrophic activity. J Nat Prod.

[147] Sommerwerk S, Kern S, Heller L, Csuk R (2014). First total synthesis of piperodione and analogs. Tetrahedron Lett.

[148] Tiwari PK, Ballav H, Aidhen IS (2018). Total synthesis of natural product piperodione and its analogues. ChemistrySelect.

[149] Harada K, Kubo M, Fukuyama Y (2020). Chemistry and neurotrophic activities of (–)-talaumidin and its derivatives. Front Chem.

[150] Wilson RM, Danishefsky SJ (2006). Application of total synthesis to problems in neurodegenereation: fascinating chemistry along the way. Acc Chem Res.

