# Correction to: The search for, and chemistry and mechanism of, neurotrophic natural products

**DOI:** 10.1007/s11418-020-01442-5

**Published:** 2020-08-20

**Authors:** Yoshiyasu Fukuyama, Miwa Kubo, Kenichi Harada

**Affiliations:** Faculty of Pharmaceutical Sciences, Tokushima Bunri University, Tokushima, 770-8514 Japan

## Correction to: Journal of Natural Medicines https://doi.org/10.1007/s11418-020-01431-8

The article The search for, and chemistry and mechanism of, neurotrophic natural products, written by Yoshiyasu Fukuyama, Miwa Kubo and Kenichi Harada, was originally published electronically on the publisher’s internet portal on 08 July 2020 without open access. With the author(s)’ decision to opt for Open Choice the copyright of the article changed on 13 July 2020 to © The Author(s) 2020 and the article is forthwith distributed under a Creative Commons Attribution 4.0 International License, which permits use, sharing, adaptation, distribution and reproduction in any medium or format, as long as you give appropriate credit to the original author(s) and the source, provide a link to the Creative Commons licence, and indicate if changes were made. The images or other third party material in this article are included in the article’s Creative Commons licence, unless indicated otherwise in a credit line to the material. If material is not included in the article’s Creative Commons licence and your intended use is not permitted by statutory regulation or exceeds the permitted use, you will need to obtain permission directly from the copyright holder. To view a copy of this licence, visit https://creativecommons.org/licenses/by/4.0.

The original article was updated.

